# Microbial Interactions within the Cheese Ecosystem and Their Application to Improve Quality and Safety

**DOI:** 10.3390/foods10030602

**Published:** 2021-03-12

**Authors:** Baltasar Mayo, Javier Rodríguez, Lucía Vázquez, Ana Belén Flórez

**Affiliations:** Departamento de Microbiología y Bioquímica, Instituto de Productos Lácteos de Asturias (IPLA), Consejo Superior de Investigaciones Científicas (CSIC), Paseo Río Linares s/n, 33300 Villaviciosa, Spain; javier.rodriguez@ipla.csic.es (J.R.); lucia.vazquez@ipla.csic.es (L.V.); abflorez@ipla.csic.es (A.B.F.)

**Keywords:** cheese, cheese microbiota, lactic acid bacteria, starters, adjunct cultures, cheese quality, cheese safety, high throughput sequencing, microbial interactions, community assembly

## Abstract

The cheese microbiota comprises a consortium of prokaryotic, eukaryotic and viral populations, among which lactic acid bacteria (LAB) are majority components with a prominent role during manufacturing and ripening. The assortment, numbers and proportions of LAB and other microbial biotypes making up the microbiota of cheese are affected by a range of biotic and abiotic factors. Cooperative and competitive interactions between distinct members of the microbiota may occur, with rheological, organoleptic and safety implications for ripened cheese. However, the mechanistic details of these interactions, and their functional consequences, are largely unknown. Acquiring such knowledge is important if we are to predict when fermentations will be successful and understand the causes of technological failures. The experimental use of “synthetic” microbial communities might help throw light on the dynamics of different cheese microbiota components and the interplay between them. Although synthetic communities cannot reproduce entirely the natural microbial diversity in cheese, they could help reveal basic principles governing the interactions between microbial types and perhaps allow multi-species microbial communities to be developed as functional starters. By occupying the whole ecosystem taxonomically and functionally, microbiota-based cultures might be expected to be more resilient and efficient than conventional starters in the development of unique sensorial properties.

## 1. General Introduction

**Figure 1:**
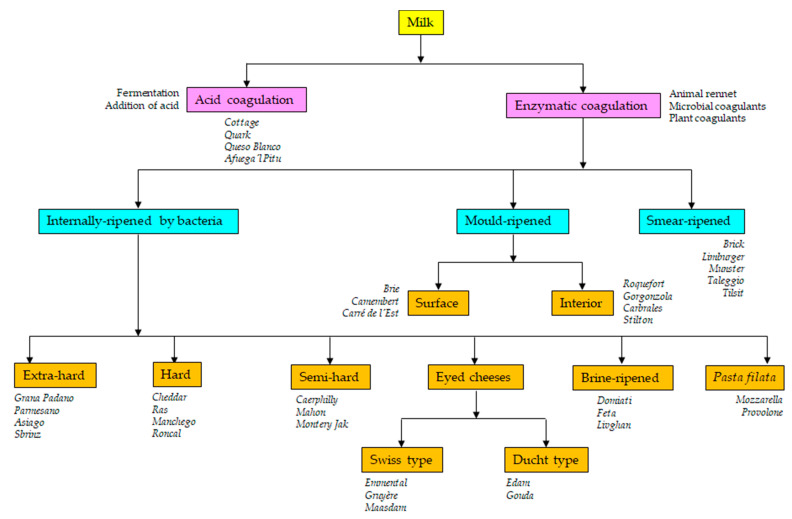
Schematic diagram of the cheese manufacturing processes and types of the resulting cheese varieties.

Cheese is a fermented milk product that dates back to Neolithic times. Traditionally, cheese was a milk-derived food that served as a means of preserving milk and its remarkable nutritive properties. Currently, the *Codex Alimentarius* defines cheese as “*a ripened or unripened, soft, semi-hard, hard, or extra-hard, dehydrated milk-derived product in which the whey protein/casein ratio does not exceed that of milk*” [1]. Thus, cheese is the generic name for a group of milk-derived food products that come in a great variety of forms, sizes, textures, aromas, and tastes. The use of milk from distinct species (cows, sheep, goats, yaks, buffalos, moose, llamas) or their mixtures and the different technological operations employed in coagulation (e.g., acidification or the addition of animal rennet- or microbial- and plant-derived coagulants), the cutting of the coagulum (from rice grain to walnut size), whey drainage, washing, heating (from 30 °C to 55 °C), pressing, salting (between 1% and 5% NaCl), ripening, dehydration, immersion (in oil, wine or brine), wrapping (with ash or flour, etc.), and the addition of spices (pepper, cumin, clover, rosemary, aromatic herbs, garlic, etc.) or colorants (chlorophylls, paprika, annatto) make cheese one of the most diverse of all foodstuffs [2,3] (Figure 1).

The sensorial properties of cheese depend on the milk type used, the feed given to the providing animal, the manufacturing practices involved, the ripening environment, the duration of ripening, and the type, numbers and activity of the microorganisms in the forming product [4,5]. Microorganisms are responsible for the fermentation of milk and for the many biochemical reactions occurring during manufacturing and ripening, which give rise to the distinctive cheese-associated textures and flavors.

In the wake of the booming amount of microbial data obtained through state-of-the-art molecular methods, this review presents updated knowledge on the composition of the cheese microbiota and summarizes the microbial interactions taking place in the cheese ecosystems. The review shows a strong focus on the biotic and abiotic factors driving the development and succession of the microbial populations and points out the potential use of this knowledge to improve the sensorial properties and safety concerns of cheese.

## 2. Cheese Starters and Adjunct Cultures

Milk can be coagulated by heating, by the formation or addition of acid, by the use of a natural coagulant (such as rennet), or a combination of these treatments (Figure 1). Spontaneous acidification is caused by the growth of lactic acid bacteria (LAB), a diverse bacterial group the members of which generate lactic acid as the main end-product of lactose fermentation. The typical LAB are arranged into the genera *Lactococcus*, *Lactobacillus*, *Leuconostoc,* and *Pediococcus* [6,7]. Via the action of complex anabolic and catabolic systems, the growth of LAB modifies the constituents of the milk (carbohydrates, proteins and lipids) [8]. These modifications do not involve nutritional or sensorial losses; rather, they increase the bioavailability and diversity of nutrients and improve the quality and complexity of flavor profiles [9]. LAB naturally present in milk or on manufacturing tools and in the environment [10,11,12,13,14,15,16] are still relied upon in many traditional fermentations [17,18,19,20,21,22,23,24,25,26,27,28]. However, improvement in milk hygiene (mainly by refrigeration and pasteurization practices) and the need for standardization have promoted the generalized use of starters [11,13], i.e., selected strains of different LAB species deliberately added to the milk to control the fermentation and standardize the quality of the fermented product (Table 1). Not surprisingly, in the search for improved starters, most microbial studies of cheese have focused on the isolation and characterization of new LAB strains of species such as *Streptococcus* (*S.*) *thermophilus*, *Lactococcus* (*Lc.*) *lactis*, *Lactobacillus* (*Lb.*) sp., and *Leuconostoc* (*Leuc.*) sp. [24,29,30,31,32,33,34,35,36,37,38]. In the industry, however, the term “starter” refers to all microorganisms added to the milk with a technological purpose, e.g., for improving the appearance, texture, and/or flavor of the final product, and thus also covers LAB species not involved in acidification, the so-called non-starter LAB (NSLAB) [39,40]. In certain cheeses, it also covers bacteria of the genera *Propionibacterium* (Emmental, Gruyère), *Brevibacterium*, and *Corynebacterium* (smear-ripened cheeses), molds and yeasts such as *Penicillium* (*P.*) *roqueforti* (blue-veined varieties), *P. camemberti* (white moldy varieties), *Geotrichum* (*G.*) *candidum*, *Debaryomyces* (*D.*) *hansenii* (moldy and smear-ripened cheeses), and others [39,40]. These secondary types of microorganisms are usually referred to as adjunct and/or ripening cultures (Table 1).

## 3. Cheese Microbiology

The microbial composition of cheese and the microbial succession of the microorganisms in the cheese matrix have traditionally been assessed by culturing methods [25,26,41,42,43,44,45,46,47,48,49]. These rely on the isolation and cultivation of microorganisms before their identification and typing. However, culturing has repeatedly been shown unreliable for the exhaustive microbial characterization of many food ecosystems [50,51,52]. For example, the selective isolation of certain microbial taxa may require unknown growth factors and/or growth conditions that are not reproduced in the laboratory media [53]. Besides, cheese can have a low pH, a reduced a_w_, and is commonly kept under harsh storage conditions (e.g., at low temperatures or in strong brine), all of which might leave certain microbes in a physiologically viable but non-cultivable state [54]. Further, microbes present in low numbers can be outcompeted in culture by numerically abundant species, impeding the effective detection of the former [45,55,56,57]. The culture techniques can therefore underestimate the microbial diversity present and sometimes even fail to detect some majority microbial groups.

**Figure 2:**
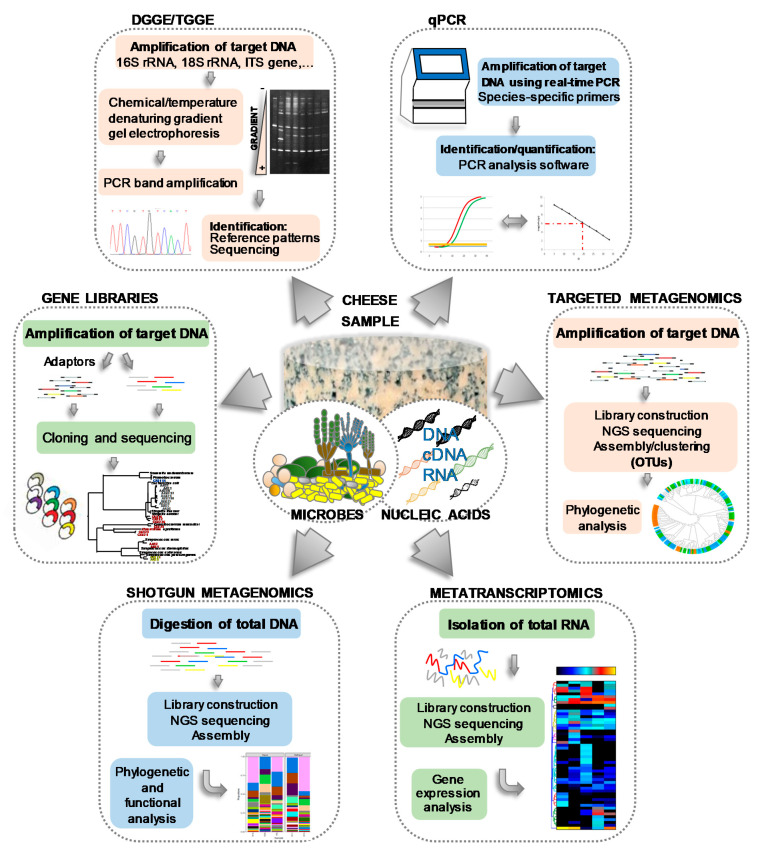
Flow chart of different microbial culture-independent molecular methods, including the main steps and final outputs, applied in cheese microbiology.

Helping to overcome the problems of culturing, numerous culture-independent, molecular methods based on the amplification of nucleic acids by polymerase chain reaction (PCR) have been developed, such as denaturing gradient gel electrophoresis (DGGE) [23,57,58,59,60,61], temporal temperature gradient electrophoresis (TTGE) [45,62], real-time quantitative PCR (qPCR) [63,64], single strand conformation polymorphism (SSCP) [65,66], the construction and analysis of gene libraries [46,66], and others [67]. The basis, similarities, differences and main outputs of such techniques, all of which have been extensively used to investigate the microbiology of cheese and dairy systems, are schematically depicted in Figure 2. As an example of the value of using such molecular techniques, *Leuc. lactis* and *Mycoplasma agalactiae,* constituting subdominant populations in two farmhouse goats’ milk cheeses and detected by PCR-TTGE, could never be recovered from cultures [45]. Similarly, although many *Arthrobacter* and *Brevibacterium* species were detected by PCR-DGGE in the smear-ripened Limburger cheese, only strains of *Arthrobacter* (*Arthr.*) *arilaitensis* and *Brevibacterium* (*Brev.*) *aurantiacum* have been retrieved by culturing [42]. Further, during the microbial typing of natural whey cultures for water-buffalo Mozzarella cheese production, *Lb. fermentum*, a majority population as judging by PCR-DGGE, was not recovered in culture [58]. Nonetheless, different LAB species have been found dominant in most cheeses both by culturing and molecular techniques, but only the latter were able to associate cheese ecosystems with occasional subdominant populations and minority microorganisms such as *Agrococcus* and *Leucobacter* [56], *Massilia* sp. [57], and *Bifidobacterium* sp. [68].

More recently, the advent of high throughput sequencing (HTS) of DNA has promoted the emergence of new, culture-independent technologies [69,70,71,72,73]. For metagenomics purposes, HTS can be used in two distinct ways: gene-specific sequencing (targeted sequencing) and the sequencing of all the microbial nucleic acids present (shotgun sequencing) (Figure 2). Compared to earlier molecular methods, HTS techniques analyze a vastly greater number of nucleic acid molecules, allowing for a much more comprehensive description of a cheese’s microbial constituents. After a pioneering use of pyrosequencing [68,74,75,76,77], Ilumina [73,78] and PacBio [79,80] technologies are currently the gold standard HTS techniques. Surprisingly, HTS has uncovered an unprecedented microbial diversity in cheeses. For example, 132 genera of the Bacteria and Archaea domains have been identified on the surface of a Swiss smear-ripened cheese [81]. Also, 238 species belonging to 14 phyla and 140 genera were recently identified in a Kazakh cheese [80], and up to 574 operational taxonomic units (OTUs) have been reported in traditional Mexican Cotija cheese [82].

LAB reads usually account for > 90% of the sequences (*Lactobacillus, Leuconostoc, Weissella, Enterococcus*, and *Lactococcus*) detected within the inner part of the cheese, but only less than 30% of reads from certain surfaces [83]. The HTS-based discovery of sequences belonging to microbes previously undetected in the dairy environment [84,85] may allow for the isolation and characterization of new biotypes by conventional [86] and novel cultivation techniques (“culturomics”) [53,87]. Further, the integration of data from culturing and culture-independent techniques (including genomics, metagenomics, metatranscriptomics, and metabolomics) is expected to provide insights into the cause–effect relationships between microbes and the metabolites that shape the sensorial descriptors of cheese, such as organic acids, fatty acids, amino acids, volatile compounds, etc. [88,89]. Certainly, while the inventory and succession of bacteria, yeasts, and molds in some cheeses are known, the functional features of the different populations are yet to be understood. By and large, most studies have been descriptive, and relatively little is known about the mechanisms that govern the architecture and dynamics of microbial populations or the molecular interactions between their members. Indeed, the activity in the cheese matrix of some uncultured/uncharacterized microbes may have a huge impact on the overall quality and safety of some cheeses [90,91]. However, understanding the technological importance and biological significance of such phenotypic microbial diversity and the genetic redundancy in cheese remains a challenge.

## 4. The Cheese Microbiota

**Figure 3:**
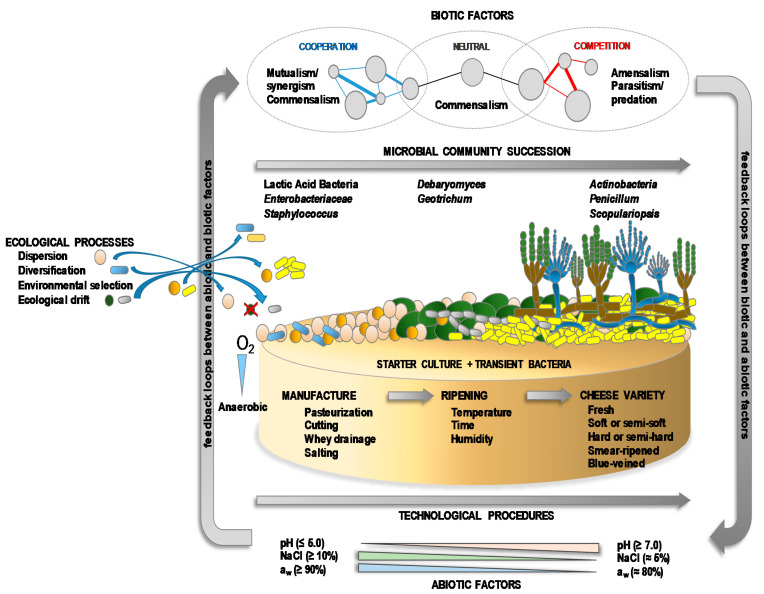
A succession of the microbial communities of the microbiota at the cheese surface, and ecological processes and environmental biotic and abiotic factors (including technological procedures) that influence microbial colonization, development and activity.

Whether fermented in a natural manner, or with the aid of starter and/or adjunct cultures, most cheeses contain a complex mixture of microbial populations—including technologically-relevant, spoilage, opportunistic and pathogenic organisms—that develops and changes throughout manufacturing and ripening [11,92,93]. All these microbes constitute the microbiota of the cheeses (Table 2). Both intrinsic (substrates, vitamins, cofactors, the presence of inhibitory/activator compounds, pH, redox potential) and extrinsic factors (oxygen availability, temperature, salt, relative humidity) drive the numbers and spatial and temporal distribution of the members of the microbiota [89,94]. The populations of the microbiota are composed of prokaryotic *Archaea* and *Bacteria*, eukaryotic yeasts and fungi [74,81,82,95,96], and viruses (mainly bacteriophages) [97,98,99]. The microbiota of cheese can be as simple as that of yogurt and other kinds of fermented milks, with perhaps just one or a very small number of LAB species present, such as in Petit-Suisse (*S. thermophilus* and *Lb. delbrueckii* subsp. *bulgaricus*) [100] and Quark (*Lc. lactis* subsp. *lactis* and *Lc. lactis* subsp. *cremoris*) [101]. More often, however, the cheese microbiota is composed of a consortium of diverse microorganisms and varies widely from one variety to another, although the dominant microbial types for each cheese type (soft, hard, natural rind, smear-ripened, blue-veined, etc.) are usually similar [42,102,103,104]. The microbiota becomes particularly complex in blue-veined and smear-ripened varieties (Table 2). As in other ecosystems, the diversity of the microbiota in cheese is governed by classical ecological processes, such as dispersion, diversification, environmental selection, and ecological drift (Figure 3). Microbial diversity and numbers are also influenced by the environmental interaction of biotic (natural fermentation, use or not of starters, presence of contaminating microbes and microbial metabolites) and abiotic factors (technological processes and environmental conditions), which modulate the implantation, development and, more importantly, the activity of the different microbes (Figure 3). Together, these variables determine the growth and function of the microorganisms and, therefore, some of the key biochemical changes they drive during ripening that lead to the unique appearance, texture, aroma and taste properties of each cheese variety, as well as their safety quality [4,8,105].

Cheese bacteria belong mainly to the phyla *Firmicutes* (LAB, enterococci, staphylococci), *Actinobacteria* (corynebacteria, propionibacteria, bifidobacteria), and *Proteobacteria* (enterobacteria) [55,56,83,104,107]. The archaeal taxa include members of *Thermocladium, Sulfurisphaera, Methanohalobium,* and others; these are minority populations (< 0.5% relative abundance) and have only ever been detected by molecular methods [57,81,82]. Among the eukaryotes, the dominant yeasts belong to the genera *Geotrichum, Debaryomyces, Kluyveromyces, Candida,* and *Yarrowia*, and the filamentous fungi are molds such as *P. camemberti, P. roqueforti* and other *Penicillium* species, which are abundant in mold-ripened cheese varieties [12,47,106,112,113]. Other filamentous fungi such as *Fusarium domesticum, Scopulariopsis* (*Sc.*) *flava* and *Sc. casei* are also found in low numbers on the surface of most cheeses [12,14,96]. Except for *P. roqueforti*, all these other mold species are only known from cheese, suggesting they are adapted (“domesticated”) to this particular habitat. In particular, *P. camemberti* derives from the wild ancestor *Penicillium commune* in a quick adaptation process that involves reduced reproductive output, reduced mycotoxin production, reduced pigmentation and, significantly, a change in the volatile compound profile from earthy to cheesy [114]. The genetic basis of this rapid “evolution” has proven to be through gene regulation instead of genome changes [114]. *Lc. lactis* subpopulations of the *lactis* and *cremoris* subspecies in dairy environments are also thought to be adapted through domestication processes [115,116]. These, and the domesticated strains of other LAB species found only in milk and dairy products, seem to have emerged recently due to the selective pressure imposed by the dairy technologies [117].

Though highly variable between varieties, the concentration of bacteria in ripened cheese may exceed 10^9^ colony forming units (cfu)/g [5,25,108,118], while those of yeasts and filamentous fungi range widely between 10^2^ and 10^7^ cfu/g [5,28,96,106,113,119]. Depending on the microbial taxon, maximum numbers are reached by the end of the fermentation (e.g., *Lc. lactis*), between day 7 to 17 (e.g., *Lactobacillus* spp.) after one to two months of ripening (e.g., filamentous fungi). Once the highest level is reached, numbers are declining slightly but consistently afterward. Variations in the composition and/or dynamics of the microbial communities making up the typical microbiota of a given cheese can lead to serious technological and sensorial defects [84,85,120,121,122] and even pose food safety risks [123,124].

## 5. Microbial Interactions in Cheese

In nature, microorganisms live in complex communities, in which different direct and indirect, cooperative and competitive microbial interactions can occur (Figure 3). Microbial interactions are mediated through a variety of molecular and physiological mechanisms, of which trophic interactions (cross-feeding) and the exchange of metabolites are the most typical. Trophic food chains enable multiple groups of organisms to survive on limited resources, increasing community diversity [125,126]. Conversely, some microbes can be inhibited or killed by metabolic substances or antimicrobial compounds produced by other components of the microbiota [127,128,129]. In general, the interactions between the different microorganisms impact the final composition and diversity of the cheese microbiota, but particularly its functionality [130,131]. In the context of milk fermentation, direct interactions refer to parasitism and apply mostly to phage-bacteria predation [132]. Under the changing environmental conditions throughout manufacture and ripening, bacteriophages are considered key players in the dynamics of the cheese microbial communities [133]. Phage predation ensures bacterial diversity by suppressing abundant strains (by the “kill the winner” theory), stabilizing the overall functionality of the host community [134]. Phages may have a tremendous effect on the fermentation, in which LAB populations need to attain high cell numbers in a very short time [132]. A fermentation failure leads usually to a subsequent improper ripening process downgrading the sensory properties of the final product. Regardless of this importance, due to the inanimate living nature of the phages, the direct bacteriophage–bacteria interactions are outside the scope of this review. Many different types of indirect interactions between the other microbial types exist [135,136], although, as in other ecosystems, the four main types in cheese involve competition, amensalism, commensalism, and mutualism [137,138,139,140].

### 5.1. Competition

In competition, two or more microorganisms compete for nutrient and energy resources in a manner that negatively affects both. The success of LAB in milk is due to their efficient use of the nutrients found in this medium, which include lactose (a rare sugar outside milk, the utilization of which requires specific transport and degradation machinery [141]), and the ability to degrade milk proteins (caseins) and efficiently take up the released amino acids and peptides [140]. Other organisms are limited by the inability of using lactose and/or the small amounts of freely available nitrogenous substrates [142,143]. Iron and zinc are also thought to be limiting micronutrients in dairy products [84]. Some microorganisms, such as *Arthrobacter, Corynebacterium*, yeasts, etc., produce siderophores to help take up these essential trace elements, while siderophore-deficient bacteria such as *Brevibacterium* and *Microbacterium*, etc., have molecular systems that help them to “steal” siderophores from their producers [84,144]. Understanding these interactions is essential, for instance, to selecting starter species and strains (or mixtures of strains) with efficient metal acquisition systems [145,146], which will allow them to strive for growth in dairy systems.

### 5.2. Amensalism

Amensalism involves interactions in which one type of microorganism negatively affects another without being affected itself. This type of relationship is commonly seen in dairy fermentations, where strains of many LAB species produce organic acids (lactic and acetic acids) that are effective inhibitors of susceptible microorganisms [128,129]. In addition to reducing the pH when released into the surrounding medium, they also have a direct inhibitory effect resulting from their undissociated forms by diffusing through the cell membranes and releasing H+ ions that acidify the cell cytoplasm [147]. Some other LAB antimicrobials, such as bacteriocins, H_2_O_2_, and fatty acids, are also thought to inhibit the growth of some organisms [148]. Bacteriocin-producing strains typically synthesize dedicated systems that protect them from these products’ harmful effects. In practice, bacteriocin-producing strains are used as “protective cultures” [149] to inhibit the development of pathogens and spoilage microorganisms in cheese. Indeed, they have been tested as inhibitors of *Listeria* (*L.*) *monocytogenes* [150,151,152,153,154,155,156], *Staph. aureus* [157,158], *Salmonella* sp. [159], *Clostridium* sp. [160,161,162], and other undesirable microbes [163]. Despite their technological use, bacteriocins may have physiological functions beyond their inhibitory activity [164,165]. Some authors [166] have suggested that subinhibitory levels may play subtle roles in guiding the succession of microbes in food fermentations.

Occasionally, the antimicrobial activity is associated with a microbial consortium rather than any single strain. For example, strong antilisterial activity exerted by some undefined consortia from the rind of smear-ripened cheeses has been repeatedly reported [167,168,169,170,171]. Via addition and erosion experiments (i.e., adding or removing one strain at a time from a mixture), attempts have been made to establish the “minimum community” showing an inhibitory property [169]. Interestingly, some minimal communities have been shown to exert greater antilisterial activity than the initial complex smear. After partial purification, an antimicrobial produced by one such minimum community proved to be a small, extremely thermo- and protease-stable molecule [168].

Certain LAB also have antifungal activity [147]. The nature and quantity of antifungal compounds produced are species- and strain-dependent. Organic acids (phenyllactic, hydroxyphenyllactic), fatty acids (decanoic, coriolic), cyclopeptides, hydrogen peroxide, and diacetyl have all been found to inhibit certain fungi [128]. The production of antifungal compounds, however, is not limited to bacteria. As such, the yeast *Williopsis saturnus* (with the killer phenotype) has been reported to inhibit the galactose-fermenting spoilage yeasts *Saccharomyces* (*Sc.*) *cerevisiae* and *Kluyveromyces* (*K.*) *marxianus* [172]. Negative yeast–yeast interactions unrelated to antimicrobials, but rather of a metabolic nature, have also been reported. In co-cultures of *D. hansenii* and *Yarrowia* (*Y.*) *lipolytica*, the latter yeast causes a shift from respiratory to fermentative metabolism in the former [173].

### 5.3. Commensalism

Commensalism occurs when a microorganism in a mixture is favored by the interactions that occur in that mixture, while other organisms experience neither negative nor positive effects. It has long been recognized that the proteolytic activity of proteinase-positive LAB cultures enables non-proteolytic species and strains to grow in milk [174,175]. The same interaction has also been reported between the LAB components of the traditional Dutch starter culture known as *Ur* [133]. In this starter, culturing and typing techniques have identified eight genetic lineages as the microbial components, including five strains of *Lc. lactis* subsp. *cremoris*, two of *Lc. lactis* subsp. *lactis* biovar *diacetylactis*, and one of *Leuc. mesenteroides*. Genome analysis and *in silico* reconstruction of metabolic maps of the different species and strains of the consortium suggested that γ-aminobutyric acid (GABA) excreted by *Lc. lactis* as part of its acid stress response might serve as a substrate for succinate formation by *Leuc. mesenteroides*. In contrast, this species did not appear to contribute to the growth of *Lc. lactis* strains [133]. Likewise, in Swiss-type cheeses, propionic acid bacteria (PAB) utilize the lactate produced by LAB, generating the typical “eyes” of these cheeses [2,4], without any apparent benefit for the latter population. The presence of lactate alone, however, cannot explain all the beneficial effects of LAB on PAB. The growth of the latter bacteria might also be enhanced by amino acids and peptides released by the LAB proteolytic system [176]. Similarly, the stimulation of LAB growth by yeasts without apparent profit of the eukaryotic microorganisms has also been reported [177,178,179]. In French *Cantalet* cheese, the use of yeasts as adjunct cultures has been found to promote the survival of *Lc. lactis* cells during ripening, and to enhance the formation of the cheese’s aroma [180]. This relationship might not be strictly commensal, however, since the yeasts might also benefit from LAB growth by using the glucose and/or galactose sugars released by some LAB species [177].

The regulation of color development in cheese rinds of a Muenster-type cheese by *Brevibacterium* (*Brev.*) *linens* via the activity of accompanying yeast species may, however, be understood as an outcome of true commensalism [181].

### 5.4. Mutualism

Mutualism is the relationship in which all the microorganisms involved benefit from their interactions. The most typical mutualistic interplay between LAB bacteria in dairy products is the so-called “protocooperation” that takes place in yogurt between *S. thermophilus* and *Lb. delbrueckii* subsp. *bulgaricus* [140,166]. This relies on casein proteolysis by *Lb. delbrueckii* subsp. *bulgaricus* via its surface caseinolytic proteinase PrtB, whose activity supplies amino acids to *S. thermophilus*. This latter bacterium, in turn, provides formic acid and carbon dioxide to the former organism [182]. Recently, it has been shown that urease activity in *S. thermophilus* is also essential in this cooperation [183]. Urease deficiency causes a shortage of ammonium and CO_2_, compounds that affect the growth of *S. thermophilus* and *Lb. delbrueckii* subsp. *bulgaricus*, respectively. Additional interactions between the two microbes might include the supply of purine precursors (xanthine, uracil) by *Lb. delbrueckii* subsp. *bulgaricus* to *S. thermophilus*, and a reduction in the formation of reactive oxygen species (ROS) by *S. thermophilus* in response to H_2_O_2_ production by *Lb. delbrueckii* subsp. *bulgaricus* via the Fenton reaction [166]. Cooperation between other LAB species, such as that seen between *Lc. lactis* and *Lb. casei* in the proteolysis of milk proteins during cheese ripening, has also been reported [184].

Cooperative cross-feeding between LAB and yeast species isolated from cheese has been abundantly described [185,186]. Yeasts can provide LAB with essential vitamins [180] or with carbon (2-oxoglutarate) and nitrogen (amino acids) sources, while LAB can provide lactic acid to non-lactose fermenting yeasts as a preferred energy substrate [187,188]. A better understanding of the metabolic activities of yeasts and LAB species and their possible interactions in cheese rind has recently been gained by combining the results of metagenomic, metatranscriptomic, and metabolomic analyses [189]. In the rind of a cheese model involving a synthetic microbiota composed of *Lc. lactis, Brev. aurantiacum, Glutamicibacter* (*G.*) *arilaitensis* (formerly *Arthr. arilaitensis*)*, Corynebacterium casei, Hafnia* (*H.*) *alvei* and *Staph. equorum*, plus the yeasts *D. hansenii, G. candidum* and *K. lactis*, several mutualistic interactions were observed [188]. *Lc. lactis* and the yeast *K. lactis*, the most active species on day one, enabled the rapid fermentation of lactose, and the lactate produced was rapidly consumed by the yeast species *D. hansenii* and *G. candidum*. The ensuing deacidification of the matrix by the yeasts allowed the ensuing development of all five acid-sensitive bacteria [189].

The biotic interactions between *D. hansenii* and strains of the acid-sensitive species *Brev. aurantiacum* and *H. alvei* have also been recently assessed in a mini-cheese model [190]. Transcriptomic profiling of the cheeses produced with different combinations of these three species revealed potential mechanisms of interaction involving iron acquisition, proteolysis, lipolysis, sulfur metabolism, and *D*-galactonate catabolism. Confirming the previous results by Dugat-Bony et al. [189], the growth of *D. hansenii* increased the pH, allowing for the development of *Brev. aurantiacum* and *H. alvei* [190]. Further, strong mutualistic interactions between the two bacteria were also observed. *Brev. aurantiacum* benefited from the production of siderophores by *H. alvei*, while *H. alvei* growth was stimulated by sulfur amino acids and other energy compounds released from casein and triglycerides via the proteases and lipases secreted by *Brev. aurantiacum* [190]. Some of these interactions are of industrial interest since proteolysis increases the pool of methionine, the substrate for the formation of volatile sulfur compounds by *H. alvei*, which increase cheese flavor.

None of the above microbial interactions rules out others occurring [135,136]. Indeed, many and complex interactions between and within the different components of the cheese microbiota surely take place at the same time throughout manufacturing and ripening. For example, commensalistic and amensalistic interactions have been observed during the investigation of the interactions between the cheese microbes *Lc. lactis, Y. lipolytica*, and *Staph. xylosus* [191]. The numbers of *Y. lipolytica* were dramatically reduced by the presence of *Staph. xylosus*, whereas, although some changes in gene expression were observed, the growth of the lactic acid bacterium was not affected by the presence of either *Staph. xylosus* or *Y. lipolytica* [191]. Similarly, LAB and adventitious non-starter organisms may compete for citrate in cheese, while cooperation in terms of proteolysis and lipolysis may occur; all these interactions can lead to increased flavor formation [192,193,194]. Growth-detrimental interactions between *S. thermophilus* and *Lb. delbrueckii* subsp. *bulgaricus* in yogurt have also been reported. Strains of either species can produce bacteriocins that inhibit those of their partner [195,196].

## 6. Dynamics of Microbial Communities in Cheese

Microbial interactions determine the development, dynamics and activity of the microbial communities that compose the cheese microbiota, which may also influence cheese quality and safety [63,192,197,198]. Microbial communities often express emergent properties that cannot be predicted based on their individual members [199]. The robustness of microbiota may also be promoted by the taxonomic, genetic and functional redundancy seen in complex microbial communities [200]. To understand the causes and consequences of the microbial interactions affecting community dynamics and functionality, strategies are required that will help identify the patterns of microorganisms that determine the processes shaping the outcomes of the microbial interactions. These strategies must also assist in unraveling the molecular mechanisms underlying the interactions [201]. In this regard, the assistance of a vast array of state-of-the-art “omic” techniques is crucial [61,202]. For example, metagenomic surveys of the microbiota of cheeses [17,74,80,82,83,95,103,107,117,118,203,204,205] can help uncover patterns of community composition, while transcriptomic profiling [173,184,189,190,191,198,206] can be used to study microorganisms in a pairwise fashion and thus dissect interaction mechanisms. Besides, metabolomic techniques [115,116,207] can be used to identify the actual chemical mediators of these interactions [85,166,189,190,208,209]. The knowledge gathered through all these techniques may provide tools for managing and manipulating the microbiota and, consequently, can contribute to cheese quality and safety [210].

It has been consistently noted that the dominant microbial populations in different cheese varieties remain the same irrespective of whether the cheese is made from raw or pasteurized milk [42,102,103,211]. This suggests that cheese-specific environmental factors allow the consistent assembly of certain microbes in each particular cheese type [201,212]. Recently, the rind microbiota of a large set of cheeses with natural, smear, and bloomy rinds, although varied and complex, has been reported to be composed of easily tractable microbial communities [205]. Intensive sampling of cheeses from Europe and the USA made at different times of year has shown the assemblage of rind microbial communities to be very consistent. More importantly, in a simple *in vitro* system (10% cheese curd agar), Wolfe et al. [205] demonstrated that the different patterns of community composition and succession in cheese rinds can be easily reconstructed from a small pool of the commonest abundant taxa (seven bacteria and four fungal species) simply by changing the inoculum size and the rind washing and drying processes followed. Moisture was found to be the best predictor of the cheese rind community’s composition. In bloomy rinds, the numbers of *Galactomyces* and four genera of highly abundant *Proteobacteria* species were found to positively correlate with the moisture level [205], while molds, *Actinobacteria*, and *Staphylococcus* species, which are all abundant in dry, natural rinds, were negatively associated with this parameter. The same research group also showed that motile bacteria from rind microbial communities (*Serratia, Halomonas, Vibrio, Psychrobacter*, and others) use the humidity associated with the physical networks created by the co-occurring filamentous fungi for dispersal [208]. The latter study highlights how fungal-mediated bacterial dispersal can promote the growth of motile organisms over that of non-motile community members. In addition to shaping the composition of the cheese rind microbiota, this interaction could have quality and safety implications, depending on whether motile microbes are of technological relevance (*H. alvei, Psychrobacter* sp., etc.) or are pathogens (*L. monocytogenes*) [208].

By analyzing the spread of three closely related *Staphylococcus* species in cheese rind biofilms, it has been shown that biotic interactions can drive the patterns of microbial species distribution [209]. Surprisingly, based on growth and competition assays in the laboratory, *Staph. equorum* (the most abundant *Staphylococcus* in cheese) proved to be a slower colonizer and weaker competitor than *Staph. xylosus* and *Staph. saprophyticus* [208]. However, *Staph. equorum* was shown to be promoted by fungi, particularly by those of the genus *Scopulariopsis* [209]. Comparative genomic and transcriptomic experiments indicated that the potential mechanism underlying this bacterium–fungus interaction was based on iron utilization. Filamentous fungi release siderophores into the cheese matrix, and the bacterium responds by overexpressing siderophore-binding proteins [209]. This reaction provides *Staph. equorum* with an exclusive and inexpensive iron source.

Nine synthetic microbial communities consisting of different strains of three bacterial species (*Staph. equorum, Brev. aurantiacum*, and *Brachybacterium alimentarium*) have been reported to show different responses to abiotic (high salt) and biotic (the presence of the fungus *Penicillium*) disturbances [213]. Some combinations of strains showed no response, while others showed a substantial shift in community composition. These differing responses were shown to correlate with differences in pigment production (light yellow to orange) and with the volatile organic compounds emitted from the rinds (nutty to sulfury) [213]. This suggests that taxonomic profiling alone may not predict well the assembly, dynamics, and functions of cheese microbiomes. However, the results stress the importance of the microbial interactions in the flavor formation in the cheese rind. Chemicals triggering the assemblage of a community do not necessarily need to be physically close to the microbial responder. Indeed, volatile compounds produced by fungi have recently been found to stimulate the growth of *Vibrio casei* [214]. The latter study showed how volatile compounds may affect the development of a microbial community in cheese and demonstrated the feasibility of using airborne chemicals to control the composition (and thus activity) of the cheese microbiota.

Together, the above studies highlight how easily tractable microbial communities within the cheese microbiota can link the results of *in vitro* experiments with in situ observations [215]. Such associations might help determine the ecological processes contributing to species distribution and the abundance of microorganisms in cheese. A difficulty in inferring hypotheses regarding the relationships of ecological processes and microbial communities and in testing them experimentally is the inability to mirror accurately under laboratory settings the natural conditions encountered during cheese manufacturing and ripening [84,189,190,208,210]. However, understanding the mechanisms behind these interactions, and the environmental conditions that induce them, is a prerequisite for engineering communities for applied purposes [197,216]. The knowledge obtained in this regard might serve to support cheesemakers’ empirical observations, such as that the use of fresh milk with low levels of psychrotrophs prevents the development of the spoilage fungus *Mucor* during ripening [217], and that lowering the humidity of the curd favors the growth of *G. candidum* while inhibiting that of *Mucor* [218]. An advantage might also be taken of biotic interactions between the typical members of the cheese microbiota to inhibit the dynamics of cheese-borne pathogens such as *L. monocytogenes* and enteropathogenic *Escherichia coli* [219].

## 7. Microbiota-Based Starters

Despite the enormous advances made in resolving microbial safety hazards and spoilage issues, the dairy industry still faces important technological challenges beyond the phage infection, such as the need for improved science-based strategies to control cheese defects including the formation of splits associated with secondary fermentation [10,121,220], the appearance of calcium lactate crystals [122], and discolorations [84,120,221]. Reducing the presence of pathogens in raw milk-made cheeses [123,221], controlling spore-formers in cheeses made from pasteurized milk [11,13,138], and reducing the production and accumulation of biogenic amines in cheese [124] also need to be pursued. However, as has been repeatedly reported [207,222,223], the outcomes produced by the raw milk microbiota in cheeses during ripening cannot be reproduced by simply adding starter and ripening cultures. One solution for developing multipurpose functional starters would be to identify, isolate and characterize competitive microorganisms within dominant and key functional populations (the so-called “core microbiota”) in each cheese type, and return them in a synthetic mixture for cheese manufacture and ripening [215,224].

Multi-species synthetic microbial communities are widely used in several biotechnological processes, as these may have properties that a single species or microbial strain alone could never show [225]. The main aim of a multi-species community culture is to occupy the ecosystem from a taxonomic viewpoint, but especially from a functional perspective [88,226]. This idea has proven successful in the inhibition of pathogens in plant roots, where, as shown above for the antilisterial activity of some cheese rind smears, the inhibition induced is deemed to be a property that emerges at the microbial community level [227]. The use of multispecies communities also shows promise for the treatment of intestinal disorders associated with microbial dysbiosis [228]; synthetic communities might soon be able to replace the unappealing treatments of fecal transplantation. To these ends, metagenomic data of the concerned ecosystem of interest can be examined by software tools [229] and in-network analyses [230,231] to search the samples for interactions between taxonomic units and biological samples. The relative abundance of biotypes, occurrence and exclusion patterns could also be scrutinized via correlation with the presence and concentration of key taste and aroma compounds [88,118]. Such analyses can help identify the core microbiota and key environmental factors that influence microbial colonization, development and activity. Using this strategy, Wang et al. [88] identified five genera as the core microbiota—*Lactobacillus, Saccharomyces, Pichia, Geotrichum*, and *Candida*—involved in the fermentation of a sorghum-derived liquor. Four yeast species (*Pichia kudriavzevii, G. candidum, Candida vini*, and *Sc. cerevisiae*) and one bacterium (*Lb. acetotolerans*) were then employed as representatives of each genus in experimental liquor manufacture. After fermentation, the synthetic mix was shown to have a flavor dynamics similar to that produced under standard conditions [88].

Synthetic microbial communities from cheese rinds containing various types of bacteria and fungi have already been used as smearing starters for the manufacture of smear-ripened cheeses [12,42,56,206]. Traditional smearing, in fact, involves an “old to young smearing” procedure, in which smears from mature cheeses dispersed in water or a saline solution are used to inoculate—as an undefined rind starter—the surface of young cheeses [39]. Some synthetic smear starters have been conceived [232] and typically contain three to six strains of deacidifying fungal species (usually *D. hansenii* and *G. candidum*) and acid-susceptible bacteria (*G. arilaitensis, Brev. aurantiacum, Brev. linens*, and/or *C. casei*). Occasionally, Gram-negative bacteria such as *H. alvei*, *Proteus vulgaris* or *Psychrobacter celer* can also be included, aiming at enhancing the production of volatile sulfur compounds [233,234]. At present, the design of such cultures is mostly empirical, and neither the biotic interactions between the different taxa involved nor the effects of abiotic factors are currently taken into account, which very commonly results in a colonization failure [145,235,236]. Scientifically sound, microbiota-based, multi-species starters composed of LAB and non-LAB species, and, if required, of eukaryotic organisms, would provide enzymatic activities that LAB alone do not possess, thus contributing to expanding the textural and flavor patterns of the cheeses produced with them. These starters might more easily resist the phage attack and reduce colonization by adventitious spoilage and pathogenic organisms.

## 8. Conclusions and Prospects

Abundant knowledge on the composition, diversity, and structure of the microbial communities in cheese has been accrued over recent decades via the use of HTS techniques. The diversity and number of species present within the microbial communities of different cheese varieties create the potential for a multitude of inter- and intra-species interactions, most of which, however, are currently unknown. Indeed, the interactions that have already been studied are limited to a few community members and a small number of exchanged metabolites. Even less is known about the molecular bases facilitating and regulating these exchanges. Neither do we have much knowledge regarding the conditions that allow the cheese microbiota to form and develop under the influence of biotic and abiotic factors, nor of how any of this translates into the improvement of cheese manufacture and ripening. As a consequence, successful cheese fermentations cannot be predicted, and technological failures of microbial origin are commonly inexplicable. In this regard, establishing chemical and/or microbial biomarkers to trace the milk fermentation would certainly be a valuable tool, which might contribute to enhancing cheese quality.

To get the most out of the omics revolution in cheesemaking, computational pipelines have to be developed to infer putative mechanisms of interaction between the many microbial populations. Constructing and using simple microbial communities in model systems might help unravel how microorganisms from complex consortia interact in their communities, and what influence they imprint on the sensorial properties of cheese. Confirmation is also required that the processes and mechanisms identified in model systems also work at the natural ecosystem scale, that is, at the cheese level. To that end, model communities should mimic natural populations as closely as possible; this will help throw light on the mechanisms involved in microbial colonization, functioning, and endurance of the different biotypes. Understanding microbial interactions of the biotypes with biotic and abiotic factors in cheese could help design strain mixtures as improved starter cultures. Knowledge of how microbes assemble into communities and the practical implications of these in cheesemaking could ultimately be used to improve the overall cheese quality and safety.

## Figures and Tables

**Table 1:** Current common species used as “starters” in industrial dairy fermentations.

Microbial Group/Species	Cheese	Type of Starter	Main Role/s
Lactic acid bacteria			
*Lc. lactis* subsp. *lactis* *Lc. lactis* subsp. *cremoris*	Most cheeses	Primary	Acidification, flavor development
*S. thermophilus* *Lb. delbrueckii* subsp. *lactis*	Italian and Swiss types	Primary	Acidification, flavor development
*Leuc. mesenteroides* subsp. *cremoris* *Leuc. lactis*	Soft and semi-hard	Secondary/adjunct	Flavor development, CO_2_ production
*Lb. helveticus*	Semi-hard, hard	Secondary/adjunct	Flavor development, health benefits
*Lb. casei/Lb. paracasei*	Artisanal	Secondary/adjunct	Flavor development
*Lb. plantarum*	Artisanal	Secondary/adjunct	Flavor development
Propionibacteria			
*Propionibacterium freudenreichii*	Swiss-type	Secondary/ripening	Hole formation, flavor development
Other bacteria			
*Brevibacterium linens*	Smear-ripened	Secondary/ripening	Color, flavor development
*Corynebacterium casei*	Smear-ripened	Secondary/ripening	Flavor development
Fungi			
*P. camemberti*	White moldy	Secondary/ripening	Aspect, texture, and flavor development
*P. roqueforti*	Blue-veined	Secondary/ripening	
*G. candidum*	Smear-ripened	Secondary/ripening	

Compiled and modified from Fox et al. [39] and Parente and Cogan [40].

**Table 2:** Non-exhaustive compilation of microbial studies of traditional cheeses, technologies applied, and dominant populations identified or detected.

Cheese/Type, Country (Milk Type)	Technique	Microbial Target	No. of Specimens	Main Families/Genera/Species (Relative Abundance); Sampling Point	Reference
Culturing					
Bryndza/soft Feta-type, Slovakia (Sheep)	Culturing	Fungi	5 species	*Geotrichum candidum* > *Kluyveromyces marxianus* > *Pichia fermentans* > *Candida inconspicua* > *Trichosporon cutaneum*	Laurencík et al. [106]
Cabrales/blue-veined, Spain (Cow, sheep, and goat)	Culturing	LAB	15 species	*Lc. lactis* subsp. *lactis* > *Lb. plantarum* > *Leuc. mesenteroides* > *Leuc. citreum* > *Enterococcus* > *Lb. paracasei*	Flórez et al. [107]
Gubbeen/smear-ripened, Ireland (Cow)	Culturing	Corynebacteria	39 species	*Corynebacterium casei* (50.2%) > *Corynebacterium mooreparkense* (26%) > *Microbacterium gubbeenense* (12.8%); cheese rind	Brennan et al. [48]
May bryndza/soft, Slovakia (Sheep)	Culturing	Bacteria Fungi	5 species 17 species	*Lc. lactis* subsp. *cremoris* > *Lc. lactis* subsp. *lactis* > *Mannheimia glucosida* *G. candidum* > *Penicillium* > *Beauveria brongniartii* > *Alternaria alternata*	Pangallo et al. [108]
Rinds of 33 cheeses/smear-ripened, various countries (Cow, sheep, or goat)	Culturing, sequencing	Microbes	104 bacterial genera, 39 fungal genera	*Staphylococcus* (78%) > *Brevibacterium* (75%) > *Corynebacterium* (75%) > *Arthrobacter* (66%) > *Lactococcus* (50%) > *Enterococcus* (41%) > *Brachybacterium* (38%) > *Microbacterium* (38%) > *Psychrobacter* (33%) > *Halomonas* (31%) > *Lactobacillus* (25%) > *Streptococcus* (22%) > *Marinilactibacillus* (22%) > *Pseudoalteromonas* (22%) > *Agrococcus* (19%) > *Micrococcus* (19%) > *Vibrio* (19%) > *Vagococcus* (16%) > *Facklamia* (16%) *Debaryomyces* (86%) > *Yarrowia* (57%) > *Candida* (54%) > *Geotrichum* (49%) > *Kluyveromyces* (32%) > *Pichia* (22%) > *Penicillium* (19%) > *Scopulariopsis* (8%) > *Fusarium* (8%)	Irlinger et al. [12]
Scamorza Altamurana/pasta filata, Italy (Cow)	Culturing	LAB	10 species	*Lb. delbrueckii* > *Streptococcus macedonicus* > *S. thermophilus* > *Enterococcus durans* > *Lb. fermentum* > *Lb. paracasei*	Baruzzi et al. [28]
Culturing and molecular methods					
Casín/kneaded, Spain (Cow)	Culturing DGGE	Bacteria	14 species	*Lc. lactis* subsp. *lactis* > *Lactococcus garvieae* > *Staphylococcus saprophyticus* > *Klebsiella* > *Lb. plantarum*	Alegría et al. [25]
		Bacteria (V1-V2 16S rDNA)	14 OTUs	*Lc. lactis, Streptococcus parauberis, S. thermophilus, Lc. garvieae, Lb. plantarum, Enterobacter, Corynebacterium variabile, Lb. paracasei, Macrococcus caseolyticus*	
Castelmagno/semi-hard, Italy (Cow)	Culturing PCR-DGGE	LAB Bacteria (V1 16S rDNA)	11 species 7 OTUs	*Lc. lactis* subsp. *lactis* > *Lb. plantarum* > *Lc. paracasei* >*Enterococcus faecium* >*E. durans* *Lb. plantarum, Lb. kefiranofaciens, Lactobacillus, Lc. lactis, Streptococcus agalactiae, M. caseolyticus*	Dolci et al. [44]
Cueva de la Magahá/hard, Spain (Goat)	Culturing PCR-TTGE	Bacteria Bacteria (V3 16S rDNA)	10 species 8 species	*Lb. paracasei* > *Lb. plantarum* > *Lb. brevis* > *Lactobacillus* > *Enterococcus* *Lb. plantarum, Lb. brevis, Lc. Lactis, S. thermophilus, Staphylococcus equorum, Lb. curvatus, Lb. paracasei*	Martín-Platero et al. [45]
Grana Padano/hard, Italy (Cow)	LH-PCR	LAB	6 species	*Lb. rhamnosus* > *Lb. paracasei* > *Lb. delbrueckii* > *Pediococcus acidilactici*	Santarelli et al. [22]
Livarot/smear-ripened, France (Cow)	Culturing Cloning	Bacteria/yeasts Bacteria (V4 16S rDNA)	8 bacteria, 5 yeasts species 8 species	*M. gubbeenense* > *Leucobacter komagatae* > *Halomonas*; cheese rind *Candida catenulata* > *Candida intermedia* > *G. candidum* > *Geotrichum* > *Yarrowia lipolytica*; cheese rind*Halomonas* > *L. komagatae* > *M. gubbeenense*; cheese rind	Mounier et al. [43]
Nottinghamshire/blue-veined, UK (Cow)	Culturing PCR-DGGE	Bacteria Bacteria (V3, V4-V5, V6-V8 16S rDNA)	12 species 11 OTUs	*Lc. lactis* subsp. *lactis* > *E. faecalis* > *Kokuria* > *Lactobacillus* *Lc. lactis, Lb. plantarum, Staph. equorum*	Yunita and Dodd [55]
Ragusano/pasta filata, Italy (Cow)	PCR-DGGE	Bacteria (V6-V8, V1-V3 16S rDNA or rRNA)	12 species	*S. thermophilus, Lb. fermentum, Lb. delbrueckii, Lc. lactis, Leuc. mesenteroides, Lb. casei, Enterococcus hirae* > *E. faecalis*	Randazzo et al. [61]
Salers/semi-hard, France (Cow)	PCR-SSCP	Bacteria (V2 16S rDNA)	9 OTUs	*E. faecium, Leuconostoc, Enterobacteriaceae, Bacillus thuringiensis, S. thermophilus, Leuc. pseudomesenteroides, Lb. pentosus, Corynebacterium variabilis, Brachybacterium nesterenkovii*	Duthoit et al. [66]
Saint Nectaire/smear-ripened, France (Cow)	Culturing SSCP-PCR	Bacteria Bacteria	21 species 12 OTUs	*Lc. lactis > Staphylococcus fleurettii* > *E. faecalis* > *S. thermophilus* > *Marinilactibacillus psychrotolerans* > *Chryseobacterium* > *Klebsiella* *Lc. lactis, S. thermophilus, Clostridium confusum, Nocardioides dubius, Arthrobacter psychrolactophilus, Enterobacter agglomerans*	Delbès et al. [46]
Molecular methods/high throughput sequencing					
Artisan cheeses/various, Ireland (Cow, goat, or sheep)	Pyrosequencing	Bacteria (V4 16S rDNA)	5 phyla 21 genera	*Lactococcus* (50–90%) > *Lactobacillus* > *Leuconostoc* > *Pseudomonas* > *Psychrobacter* > *Staphylococcus* > *Arthrobacter > Faecalibacterium*; common to 62 cheeses	Quigley et al. [76]
Buryatian/soft, Kazakhstan (Cow)	PacBio sequencing	Microbes	7 phyla, 82 genera, 145 species	*Lactococcus* (51.46%) > *Streptococcus* (17.81%) > *Pseudomonas* (5.48%) > *Acetobacter* (4.83%) > *Klebsiella* (3.36%) > *Lactobacillus* (2.36%) > *Acinetobacter* (1.84%) > *Raoultella* (1.63%)	Jin et al. [79]
Canestrato Pugliese/hard, Italy (Sheep)	Pyrosequencing	Bacteria (V1-V3 16S rDNA)	28 genera	*Lactococcus* (87.2%) > *Lactobacillus* (4.8%; mainly *Lb. plantarum* and *Lb. sakei*) > *Leuconostoc* (3.9%)	De Pasquale et al. [109]
Cheddar/semi-hard, UK (Cow)	Illumina sequencing	Bacteria (V4 16S rDNA)	159 OTUs	*Streptococcus* > *Lactococcus* > *Lactobacillus* > *Staphylococcus* (70%); interior	Afshari et al. [110]
Gouda-like cheese/semi-hard, USA (Cow)	Illumina sequencing	Bacteria (V4 16S rDNA)	36 genera	*Bacillaceae* > *Lactococcus* > *Lactobacillus* > *Streptococcus* > *Staphylococcus*	Salazar et al. [111]
Cotija/hard, Mexico (Cow)	Illumina sequencing	Microbes	31 phyla, 574 genera	*Lb. plantarum* > *Leuc. mesenteroides* > *Weissella paramesenteroides* (>80%) *Aerococcus* > *Enterococcus* > *Lactococcus* > *Staphylococcus* (<10%)	Escobar-Zepeda et al. [82]
Grana/hard, Italy (Cow)	RT-PCR-DGGE Pyrosequencing	Bacteria (V1 16S rDNA) Bacteria (V1-V3 16S rDNA)	16 OTUs 25 genera	*Lb. helveticus, Lb. delbrueckii, S. thermophilus, Lb. acidophilus, Lb. rhamnosus, Acetobacter baumanii, Propionibacterium* *Lb. helveticus* > *Propionibacterium* > *Lb. delbrueckiii* > *Lb. casei* > *Lb. rhamnosus* > *S. thermophilus* > *Staphylococcus* > *Lb. brevis*	Alessandria et al. [95]
Artisanal cheeses/soft, Kazakhstan (Cow)	PacBio sequencing	Microbes	14 phyla, 140 genera, 238 species	*Lc. lactis* (28.93%) > *Lb. helveticus* (26.43%) > *S. thermophilus* (12.18%) > *Lb. delbrueckii* (12.15%)	Li et al. [80]
Ocosingo/semi-hard, Mexico (Cow)	Pyrosequencing	Bacteria (V1 16S rDNA)	162 OTUs	*S. thermophilus* > *Lc. lactis* > *Lb. helveticus* > *Lb. delbrueckii* > *Lb. plantarum* (70%); interior	Aldrete-Tapia et al. [17]
Tomme d’Orchies/semi-hard, France (Cow)	Illumina sequencing	Bacteria (V1-V3 16S rDNA)	10 species core, 21 species surface	*Lactococcuss* > *Streptococcus* (66%); interior *Lactobacillus* > *Lactococcus* > *Corynebacterium* > *Micrococcales* > *Psychrobacter* (80%); surface	Ceugniez et al. [83]
Tomme d’Orchies/semi-hard, France (Cow)	Illumina sequencing	Fungi 5.8S-ITS2	30 OTUs	*Y. lipolytica* > *G. candidum*/*Galactomyces geotrichum* (99%); interior *Y. lipolytica* > *G. candidum*/*Galactomyces geotrichum* (98%); surface	Ceugniez et al. [112]

## References

[1] Codex Standard 283-1978 (2013). Codex General Standard for Cheese.

[2] Fox P.F., Guinee T.P., Cogan T.M., McSweeney P.L.H., Fox P.F., Guinee T.P., Cogan T.M., McSweeney P.L.H. (2017). Principal families of cheese. Fundamentals of Cheese Science.

[3] Kosikowski F.V., Mistry V.V., Kosikowski F.V. (1997). Cheese and Fermented Milk Foods.

[4] Fox P.F., Guinee T.P., Cogan T.M., McSweeney P.L.H., Fox P.F., Guinee T.P., Cogan T.M., McSweeney P.L.H. (2017). Microbiology of cheese ripening. Fundamentals of Cheese Science.

[5] Montel M.C., Buchin S., Mallet A., Delbes-Paus C., Vuitton D.A., Desmasures N., Berthier F. (2014). Traditional cheeses: Rich and diverse microbiota with associated benefits. Int. J. Food Microbiol..

[6] Bintis T. (2018). Lactic acid bacteria as starter cultures: An update in their metabolism and genetics. AIMS Microbiol..

[7] Carr F.J., Chill D., Maida N. (2002). The lactic acid bacteria: A literature survey. Crit. Rev. Microbiol..

[8] Smit G., Smit B.A., Engels W.J. (2005). Flavour formation by lactic acid bacteria and biochemical flavour profiling of cheese products. FEMS Microbiol. Rev..

[9] Fardet A., Dupont D., Rioux L.E., Turgeon S.L. (2019). Influence of food structure on dairy protein, lipid and calcium bioavailability: A narrative review of evidence. Crit. Rev. Food Sci. Nutr..

[10] Metzger S.A., Hernandez L.L., Suen G., Ruegg P.L. (2018). Understanding the milk microbiota. Vet. Clin. North Am. Food Anim. Pract..

[11] Boor K.J., Wiedmann M., Murphy S., Alcaine S. (2017). A 100-Year Review: Microbiology and safety of milk handling. J. Dairy Sci..

[12] Irlinger F., Layec S., Hélinck S., Dugat-Bony E. (2015). Cheese rind microbial communities: Diversity, composition and origin. FEMS Microbiol. Lett..

[13] Quigley L., O’Sullivan O., Stanton C., Beresford T.P., Ross R.P., Fitzgerald G.F., Cotter P.D. (2013). The complex microbiota of raw milk. FEMS Microbiol. Rev..

[14] Lavoie K., Touchette M., St-Gelais D., Labrie S. (2012). Characterization of the fungal microflora in raw milk and specialty cheeses of the province of Quebec. Dairy Sci. Technol..

[15] Mounier J., Goerges S., Gelsomino R., Vancanneyt M., Vandemeulebroecke K., Hoste B., Brennan N.M., Scherer S., Swings J., Fitzgerald G.F. (2006). Sources of the adventitious microflora of a smear-ripened cheese. J. Appl. Microbiol..

[16] Desmasures N., Bazin F., Guéguen M. (1997). Microbiological composition of raw milk from selected farms in the Camembert region of Normandy. J. Appl. Microbiol..

[17] Aldrete-Tapia A., Escobar-Ramírez C.M., Tamplin M.L., Hernández-Iturriaga M. (2018). Characterization of bacterial communities in Mexican artisanal raw milk "Bola de Ocosingo" cheese by high-throughput sequencing. Front. Microbiol..

[18] Frétin M., Martin B., Rifa E., Isabelle V.M., Pomiès D., Ferlay A., Montel M.C., Delbès C. (2018). Bacterial community assembly from cow teat skin to ripened cheeses is influenced by grazing systems. Sci. Rep..

[19] Haastrup M.K., Johansen P., Malskær A.H., Castro-Mejía J.L., Kot W., Krych L., Arneborg N., Jespersen L. (2018). Cheese brines from Danish dairies reveal a complex microbiota comprising several halotolerant bacteria and yeasts. Int. J. Food Microbiol..

[20] Lucchini R., Cardazzo B., Carraro L., Negrinotti M., Balzan S., Novelli E., Fasolato L., Fasoli F., Farina G. (2018). Contribution of natural milk culture to microbiota, safety and hygiene of raw milk cheese produced in alpine malga. Ital. J. Food Saf..

[21] Quijada N.M., Mann E., Wagner M., Rodríguez-Lázaro D., Hernández M., Schmitz-Esser S. (2018). Autochthonous facility-specific microbiota dominates washed-rind Austrian hard cheese surfaces and its production environment. Int. J. Food Microbiol..

[22] Santarelli M., Bottari B., Lazzi C., Neviani E., Gatti M. (2013). Survey on the community and dynamics of lactic acid bacteria in Grana Padano cheese. Syst. Appl. Microbiol..

[23] Edalatian M.R., Habibi M.B., Mortazavi S.A., Alegría A., Nassiri M.R., Bassam M.R., Mayo B. (2012). Microbial diversity of the traditional Iranian cheeses Lighvan and Koozeh, as revealed by polyphasic culturing and culture-independent approaches. Dairy Sci. Technol..

[24] Feutry F., Oneca M., Berthier F., Torre P. (2012). Biodiversity and growth dynamics of lactic acid bacteria in artisanal PDO Ossau-Iraty cheeses made from raw ewe’s milk with different starters. Food Microbiol..

[25] Alegría A., Álvarez-Martín P., Sacristán N., Fernández E., Delgado S., Mayo B. (2009). Diversity and evolution of majority microbial populations during manufacturing and ripening of Casín, a Spanish traditional, starter-free cheese made of raw cow’s milk. Int. J. Food Microbiol..

[26] Callon C., Berdagué J.L., Dufour E., Montel M.C. (2005). The effect of raw milk microbial flora on the sensory characteristics of Salers-type cheeses. J. Dairy Sci..

[27] Morales P., Fernández-García E., Gaya P., Núñez M. (2003). Formation of volatile compounds by wild *Lactococcus lactis* strains isolated from raw ewes’ milk cheese. Int. Dairy J..

[28] Baruzzi F., Matarante A., Morea M., Cocconcelli P.S. (2002). Microbial community dynamics during the Scamorza Altamurana cheese natural fermentation. J. Dairy Sci..

[29] Moser A., Schafroth K., Meile L., Egger L., Badertscher R., Irmler S. (2018). Population dynamics of *Lactobacillus helveticus* in Swiss Gruyère-type cheese manufactured with natural whey cultures. Front. Microbiol..

[30] Castro R.D., Oliveira L.G., Sant’Anna F.M., Luiz L.M.P., Sandes S.H.C., Silva C.I.F., Silva A.M., Nunes A.C., Penna C.F.A.M., Souza M.R. (2016). Lactic acid microbiota identification in water, raw milk, endogenous starter culture, and fresh Minas artisanal cheese from the Campo das Vertentes region of Brazil during the dry and rainy seasons. J. Dairy Sci..

[31] Solieri L., Bianchi A., Giudici P. (2012). Inventory of non-starter lactic acid bacteria from ripened Parmigiano Reggiano cheese as assessed by a culture dependent multiphasic approach. Syst. Appl. Microbiol..

[32] Nieto-Arribas P., Seseña S., Poveda J.M., Palop L., Cabezas L. (2010). Genotypic and technological characterization of *Leuconostoc* isolates to be used as adjunct starters in Manchego cheese manufacture. Food Microbiol..

[33] van Hoorde K., van Leuven I., Dirinck P., Heyndrickx M., Coudijzer K., Vandamme P., Huys G. (2010). Selection, application and monitoring of *Lactobacillus paracasei* strains as adjunct cultures in the production of Gouda-type cheeses. Int. J. Food Microbiol..

[34] Henri-Dubernet S., Desmasures N., Guéguen M. (2008). Diversity and dynamics of lactobacilli populations during ripening of RDO Camembert cheese. Can. J. Microbiol..

[35] Sánchez I., Seseña S., Poveda J.M., Cabezas L., Palop L. (2006). Genetic diversity, dynamics, and activity of *Lactobacillus* community involved in traditional processing of artisanal Manchego cheese. Int. J. Food Microbiol..

[36] Dasen A., Berthier F., Grappin R., Williams A.G., Banks J. (2003). Genotypic and phenotypic characterization of the dynamics of the lactic acid bacterial population of adjunct-containing Cheddar cheese manufactured from raw and microfiltered pasteurised milk. J. Appl. Microbiol..

[37] Corroler D., Mangin I., Desmasures N., Gueguen M. (1998). An ecological study of lactococci isolated from raw milk in the camembert cheese registered designation of origin area. Appl. Environ. Microbiol..

[38] Desmasures N., Mangin I., Corroler D., Guéguen M. (1998). Characterization of lactococci isolated from milk produced in the Camembert region of Normandy. J. Appl. Microbiol..

[39] Fox P.F., Guinee T.P., Cogan T.M., McSweeney P.L.H., Fox P.F., Guinee T.P., Cogan T.M., McSweeney P.L.H. (2017). Starter cultures. Fundamentals of Cheese Science.

[40] Parente E., Cogan T.M., Fox P.O. (2004). Starter cultures: General aspects. Cheese: Chemistry, Physics and Microbiology.

[41] Giello M., La Storia A., Masucci F., Di Francia A., Ercolini D., Villani F. (2017). Dynamics of bacterial communities during manufacture and ripening of traditional Caciocavallo of Castelfranco cheese in relation to cows’ feeding. Food Microbiol..

[42] Cogan T.M., Goerges S., Gelsomino R., Larpin S., Hohenegger M., Bora N., Jamet E., Rea M.C., Mounier J., Vancanneyt M. (2014). Biodiversity of the surface microbial consortia from Limburger, Reblochon, Livarot, Tilsit, and Gubbeen cheeses. Microbiol. Spectr..

[43] Mounier J., Monnet C., Jacques N., Antoinette A., Irlinger F. (2009). Assessment of the microbial diversity at the surface of Livarot cheese using culture-dependent and independent approaches. Int. J. Food Microbiol..

[44] Dolci P., Alessandria V., Rantsiou K., Rolle L., Zeppa G., Cocolin L. (2008). Microbial dynamics of Castelmagno PDO, a traditional Italian cheese, with a focus on lactic acid bacteria ecology. Int. J. Food Microbiol..

[45] Martín-Platero A.M., Valdivia E., Maqueda M., Martín-Sánchez I., Martínez-Bueno M. (2008). Polyphasic approach to bacterial dynamics during the ripening of Spanish farmhouse cheese, using culture-dependent and -independent methods. Appl. Environ. Microbiol..

[46] Delbès C., Ali-Mandjee L., Montel M.C. (2007). Monitoring bacterial communities in raw milk and cheese by culture-dependent and -independent 16S rRNA gene-based analyses. Appl. Environ. Microbiol..

[47] Rea M.C., Görges S., Gelsomino R., Brennan N.M., Mounier J., Vancanneyt M., Scherer S., Swings J., Cogan T.M. (2007). Stability of the biodiversity of the surface consortia of Gubbeen, a red-smear cheese. J. Dairy Sci..

[48] Brennan N.M., Ward A.C., Beresford T.P., Fox P.F., Goodfellow M., Cogan T.M. (2002). Biodiversity of the bacterial flora on the surface of a smear cheese. Appl. Environ. Microbiol..

[49] Callon C., Millet L., Montel M.C. (2004). Diversity of lactic acid bacteria isolated from AOC Salers cheese. J. Dairy Res..

[50] Ercolini D. (2013). High-throughput sequencing and metagenomics: Moving forward in the culture-independent analysis of food microbial ecology. Appl. Environ. Microbiol..

[51] Quigley L., O’Sullivan O., Beresford T.P., Ross R.P., Fitzgerald G.F., Cotter P.D. (2011). Molecular approaches to analysing the microbial composition of raw milk and raw milk cheese. Int. J. Food Microbiol..

[52] Jany J.L., Barbier G. (2008). Culture-independent methods for identifying microbial communities in cheese. Food Microbiol..

[53] Overmann J., Abt B., Sikorski J. (2017). Present and future of culturing bacteria. Ann. Rev. Microbiol..

[54] Kumar S.S., Ghosh A.R. (2019). Assessment of bacterial viability: A comprehensive review on recent advances and challenges. Microbiology.

[55] Yunita D., Dodd C.E.R. (2018). Microbial community dynamics of a blue-veined raw milk cheese from the United Kingdom. J. Dairy Sci..

[56] Larpin-Laborde S., Imran M., Bonaïti C., Bora N., Gelsomino R., Goerges S., Irlinger F., Goodfellow M., Ward A.C., Vancanneyt M. (2011). Surface microbial consortia from Livarot, a French smear-ripened cheese. Can. J. Microbiol..

[57] Feligini M., Panelli S., Buffoni J.N., Bonacina C., Andrighetto C., Lombardi A. (2012). Identification of microbiota present on the surface of Taleggio cheese using PCR-DGGE and RAPD-PCR. J. Food Sci..

[58] Ercolini D., Moschetti G., Blaiotta G., Coppola S. (2001). The potential of a polyphasic PCR-DGGE approach in evaluating microbial diversity of natural whey cultures for water-buffalo Mozzarella cheese production: Bias of culture-dependent and culture-independent analyzes. Syst. Appl. Microbiol..

[59] Dolci P., Zenato S., Pramotton R., Barmaz A., Alessandria V., Rantsiou K., Cocolin L. (2013). Cheese surface microbiota complexity: RT-PCR-DGGE, a tool for a detailed picture?. Int. J. Food Microbiol..

[60] Flórez A.B., Mayo B. (2006). Microbial diversity and succession during the manufacture and ripening of traditional, Spanish, blue-veined Cabrales cheese, as determined by PCR-DGGE. Int. J. Food Microbiol..

[61] Randazzo C.L., Torriani S., Akkermans A.D., de Vos W.M., Vaughan E.E. (2002). Diversity, dynamics, and activity of bacterial communities during production of an artisanal Sicilian cheese as evaluated by 16S rRNA analysis. Appl. Environ. Microbiol..

[62] Roth E., Miescher Schwenninger S., Hasler M., Eugster-Meier E., Lacroix C. (2010). Population dynamics of two antilisterial cheese surface consortia revealed by temporal temperature gradient gel electrophoresis. BMC Microbiol..

[63] Bertani G., Levante A., Lazzi C., Bottari B., Gatti M., Neviani E. (2020). Dynamics of a natural bacterial community under technological and environmental pressures: The case of natural whey starter for Parmigiano Reggiano cheese. Food Res. Int..

[64] Agrimonti C., Bottari B., Sardaro M.L.S., Marmiroli N. (2019). Application of real-time PCR (qPCR) for characterization of microbial populations and type of milk in dairy food products. Crit. Rev. Food Sci. Nutr..

[65] Hermet A., Mounier J., Keravec M., Vasseur V., Barbier G., Jany J.L. (2014). Application of capillary electrophoresis single-stranded conformation polymorphism (CE-SSCP) analysis for identification of fungal communities in cheese. Food Microbiol..

[66] Duthoit F., Godon J.J., Montel M.C. (2003). Bacterial community dynamics during production of registered designation of origin Salers cheese as evaluated by 16S rRNA gene single-strand conformation polymorphism analysis. Appl. Environ. Microbiol..

[67] Sánchez J.I., Rossetti L., Martínez B., Rodríguez A., Giraffa G. (2006). Application of reverse transcriptase PCR-based T-RFLP to perform semi-quantitative analysis of metabolically active bacteria in dairy fermentations. J. Microbiol. Methods.

[68] Alegría A., Szczesny P., Mayo B., Bardowski J., Kowalczyk M. (2012). Biodiversity in Oscypek, a traditional Polish cheese, determined by culture-dependent and -independent approaches. Appl. Environ. Microbiol..

[69] Afshari R., Pillidge C.J., Dias D.A., Osborn A.M., Gill H. (2020). Cheesomics: The future pathway to understanding cheese flavour and quality. Crit. Rev. Food Sci. Nutr..

[70] Cao Y., Fanning S., Proos S., Jordan K., Srikumar S. (2017). A review on the applications of Next Generation Sequencing technologies as applied to food-related microbiome studies. Front. Microbiol..

[71] Kergourlay G., Taminiau B., Daube G., Champomier Vergès M.C. (2015). Metagenomic insights into the dynamics of microbial communities in food. Int. J. Food Microbiol..

[72] Mayo B., Rachid C.T.C.C., Alegría A., Leite A.M.O., Peixoto R.S., Delgado S. (2014). Impact of next generation sequencing techniques in Food Microbiology. Curr. Genom..

[73] Bokulich N.A., Mills D.A. (2012). Next-generation approaches to the microbial ecology of food fermentations. BMB Rep..

[74] Ercolini D., De Filippis F., La Storia A., Iacono M. (2012). “Remake” by high-throughput sequencing of the microbiota involved in the production of water buffalo Mozzarella cheese. Appl. Environ. Microbiol..

[75] Lusk T.S., Ottensen A.R., White J.R., Allard M.W., Brown E.W., Kase J.E. (2012). Characterization of microflora in Latin-style cheeses by next-generation sequencing technology. BCM Microbiol..

[76] Quigley L., O’Sullivan O., Beresford T.P., Ross R.P., Fitzgerald G.F., Cotter P.D. (2012). High-throughput sequencing for detection of subpopulations of bacteria not previously associated with artisanal cheeses. Appl. Environ. Microbiol..

[77] Masoud W., Takamiya M., Vogensen F.K., Lillevang S., Al-Soud W.A., Sørensen S.J., Jakobsen M. (2010). Characterization of bacterial populations in Danish raw milk cheeses made with different starter cultures by denaturing gradient gel electrophoresis and pyrosequencing. Int. Dairy J..

[78] Yeluri Jonnala B.R., McSweeney P.L.H., Sheehan J.J., Cotter P.D. (2018). Sequencing of the cheese microbiome and its relevance to industry. Front. Microbiol..

[79] Jin H., Mo L., Pan L., Hou Q., Li C., Darima I., Yu J. (2018). Using PacBio sequencing to investigate the bacterial microbiota of traditional Buryatian cottage cheese and comparison with Italian and Kazakhstan artisanal cheeses. J. Dairy Sci..

[80] Li J., Zheng Y., Xu H., Xi X., Hou Q., Feng S., Wuri L., Bian Y., Yu Z., Kwok L.Y. (2017). Bacterial microbiota of Kazakhstan cheese revealed by single molecule real time (SMRT) sequencing and its comparison with Belgian, Kalmykian and Italian artisanal cheeses. BMC Microbiol..

[81] Ritschard J.S., Amato L., Kumar Y., Müller B., Meile L., Schuppler M. (2018). The role of the surface smear microbiome in the development of defective smear on surface-ripened red-smear cheese. AIMS Microbiol..

[82] Escobar-Zepeda A., Sanchez-Flores A., Quirasco Baruch M. (2016). Metagenomic analysis of a Mexican ripened cheese reveals a unique complex microbiota. Food Microbiol..

[83] Ceugniez A., Taminiau B., Coucheney F., Jacques P., Delcenserie V., Daube G., Drider D. (2017). Use of a metagenetic approach to monitor the bacterial microbiota of “Tomme d’Orchies” cheese during the ripening process. Int. J. Food Microbiol..

[84] Cleary J.L., Kolachina S., Wolfe B.E., Sanchez L.M. (2018). Coproporphyrin III produced by the bacterium *Glutamicibacter arilaitensis* binds zinc and is upregulated by fungi in cheese rinds. mSystems.

[85] Kamelamela N., Zalesne M., Morimoto J., Robbat A., Wolfe B.E. (2018). Indigo- and indirubin-producing strains of *Proteus* and *Psychrobacter* are associated with purple rind defect in a surface-ripened cheese. Food Microbiol..

[86] Delgado D., Rachid C.T.C.C., Fernández E., Rychlik T., Alegría A., Peixoto R.S., Mayo B. (2013). Diversity of thermophilic bacteria in raw, pasteurized and selectively-cultured milk, as assessed by culturing, PCR-DGGE and pyrosequencing. Food Microbiol..

[87] Ito T., Skizuka T., Kishi N., Yamahita A., Kuroda M. (2018). Conventional culture methods with commercially available media unveil the presence of novel culturable bacteria. Gut Microbes.

[88] Wang S., Wu Q., Nie Y., Wu J., Xu Y. (2019). Construction of a synthetic microbiota for reproducible flavor compound metabolism in Chinese light-aroma-type liquor produced by solid-state fermentation. Appl. Environ. Microbiol..

[89] De Pasquale I., Di Cagno R., Buchin S., De Angelis M., Gobbetti M. (2016). Spatial distribution of the metabolically active microbiota within Italian PDO ewes’ milk cheeses. PLoS ONE.

[90] Almeida M., Hébert A., Abraham A.L., Rasmussen S., Monnet C., Pons N., Delbès C., Loux V., Batto J.M., Leonard P. (2014). Construction of a dairy microbial genome catalog opens new perspectives for the metagenomic analysis of dairy fermented products. BMC Genom..

[91] Bonaïti C., Irlinger F., Spinnler H.E., Engel E. (2005). An iterative sensory procedure to select odor-active associations in complex consortia of microorganisms: Application to the construction of a cheese model. J. Dairy Sci..

[92] Mayo B., Ammor M.S., Delgado S., Alegría A., Tmang J.P., Kilasapathy K. (2010). Fermented milk products. Fermented Food and Beverages of the World.

[93] Wouters J.T.M., Ayad E.H.E., Hugenholtz J., Smit G. (2002). Microbes from raw milk for fermented dairy products. Int. Dairy J..

[94] Sheedan A., O’Cuinn G., Fitzgerald R.J., Wilkinson M.G. (2009). Distribution of microbial flora, intracellular enzymes and compositional indices throughout a 12 kg Cheedar cheese block during ripening. Int. Dairy J..

[95] Alessandria V., Ferrocino I., De Filippis F., Fontana M., Rantsiou K., Ercolini D., Cocolin L. (2016). Microbiota of an Italian Grana-like cheese during manufacture and ripening, unraveled by 16S rRNA-based approaches. Appl. Environ. Microbiol..

[96] Ropars J., Cruaud C., Lacoste S., Dupont J. (2012). A taxonomic and ecological overview of cheese fungi. Int. J. Food Microbiol..

[97] Oliveira J., Mahony J., Hanemaaijer L., Kouwen T.R.H.M., van Sinderen D. (2018). Biodiversity of bacteriophages infecting *Lactococcus lactis* starter cultures. J. Dairy Sci..

[98] Alexeeva S., Guerra-Martínez J.A., Spus M., Smid E.J. (2018). Spontaneously induced prophages are abundant in a naturally evolved bacterial starter culture and deliver competitive advantage to the host. BMC Microbiol..

[99] Mahony J., Moscarelli A., Kelleher P., Lugli G.A., Ventura M., Settanni L., van Sinderen D. (2017). Phage biodiversity in artisanal cheese wheys reflects the complexity of the fermentation process. Viruses.

[100] Cardarelli H.R., Saad S.M., Gibson G.R., Vulevic J. (2007). Functional petit-suisse cheese: Measure of the prebiotic effect. Anaerobe.

[101] Ferreiro T., Martínez S., Gayoso L., Rodríguez-Otero J.L. (2016). 2016. Evolution of phospholipid contents during the production of quark cheese from buttermilk. J. Dairy Sci..

[102] Lee J., Seo Y., Ha J., Kim S., Choi Y., Oh H., Lee Y., Kim Y., Kang J., Park E. (2020). Influence of milk microbiota on *Listeria monocytogenes* survival during cheese ripening. Food Sci. Nutr..

[103] Delcenserie V., Taminiau B., Delhalle L., Nezer C., Doyen P., Crevecoeur S., Roussey D., Korsak N., Daube G. (2014). Microbiota characterization of a Belgian protected designation of origin cheese, Herve cheese, using metagenomic analysis. J. Dairy Sci..

[104] Fuka M.M., Wallisch S., Engel M., Welzl G., Havranek J., Schloter M. (2013). Dynamics of bacterial communities during the ripening process of different Croatian cheese types derived from raw ewe’s milk cheeses. PLoS ONE.

[105] Fox P.F., Guinee T.P., Cogan T.M., McSweeney P.L.H., Fox P.F., Guinee T.P., Cogan T.M., McSweeney P.L.H. (2017). Biochemistry of cheese ripening. Fundamentals of Cheese Science.

[106] Laurencík M., Sulo P., Sláviková E., Piecková E., Seman M., Ebringer L. (2008). The diversity of eukaryotic microbiota in the traditional Slovak sheep cheese-bryndza. Int. J. Food Microbiol..

[107] Flórez A.B., Álvarez-Martín P., López-Díaz T.M., Mayo B. (2006). Microbiological characterisation of the traditional Spanish blue-veined Cabrales cheese: Identification of dominant lactic acid bacteria. Eur. Food Res. Technol..

[108] Pangallo D., Saková N., Koreňová J., Puškárová A., Kraková L., Valík L., Kuchta T. (2014). Microbial diversity and dynamics during the production of May bryndza cheese. Int. J. Food Microbiol..

[109] De Pasquale I., Calasso M., Mancini L., Ercolini D., La Storia A., De Angelis M., Di Cagno R., Gobbetti M. (2014). Causal relationship between microbial ecology dynamics and proteolysis during manufacture and ripening of protected designation of origin (PDO) cheese Canestrato Pugliese. Appl. Environ. Microbiol..

[110] Afshari R., Pillidge C.J., Read E., Rochfort S., Dias D.A., Osborn A.M., Gill H. (2020). New insights into cheddar cheese microbiota-metabolome relationships revealed by integrative analysis of multi-omics data. Sci. Rep..

[111] Salazar J.K., Carstens C.K., Ramachandran P., Shazer A.G., Narula S.S., Reed E., Ottesen A., Schill K.M. (2018). Metagenomics of pasteurized and unpasteurized Gouda cheese using targeted 16S rDNA sequencing. BMC Microbiol..

[112] Ceugniez A., Taminiau B., Coucheney F., Jacques P., Delcenserie V., Daube G., Drider D. (2017). Fungal diversity of “Tomme d’Orchies” cheese during the ripening process as revealed by a metagenomic study. Int. J. Food Microbiol..

[113] Ceugniez A., Drider D., Jacques P., Coucheney F. (2015). Yeast diversity in a traditional French cheese “Tomme d’orchies” reveals infrequent and frequent species with associated benefits. Food Microbiol..

[114] Bodinaku I., Shaffer J., Connors A.B., Steenwyk J.L., Biango-Daniels M.N., Kastman E.K., Rokas A., Robbat A., Wolfe B.E. (2019). Rapid phenotypic and metabolomic domestication of wild *Penicillium* molds on cheese. mBio.

[115] Laroute V., Tormo H., Couderc C., Mercier-Bonin M., Le Bourgeois P., Cocaign-Bousquet M., Daveran-Mingot M.L. (2017). From genome to phenotype: An integrative approach to evaluate the biodiversity of *Lactococcus lactis*. Microorganisms.

[116] Cavanagh D., Fitzgerald G.F., McAuliffe O. (2015). From field to fermentation: The origins of *Lactococcus lactis* and its domestication to the dairy environment. Food Microbiol..

[117] Douglas G.L., Klaenhammer T.R. (2010). Genomic evolution of domesticated microorganisms. Annu. Rev. Food Sci. Technol..

[118] Zheng X., Liu F., Shi X., Wang B., Li K., Li B., Zhuge B. (2018). Dynamic correlations between microbiota succession and flavor development involved in the ripening of Kazak artisanal cheese. Food Res. Int..

[119] Flórez A.B., Álvarez-Martín P., López-Díaz T.M., Mayo B. (2007). Morphotypic and molecular identification of filamentous fungi from Spanish blue-veined Cabrales cheese and technological characterisation of *Penicillium roqueforti* and *Geotrichum candidum* strains. Int. Dairy J..

[120] Guzzon R., Carafa I., Tuohy K., Cervantes G., Vernetti L., Barmaz A., Larcher R., Franciosi E. (2017). Exploring the microbiota of the red-brown defect in smear-ripened cheese by 454-pyrosequencing and its prevention using different cleaning systems. Food Microbiol..

[121] Bassi D., Puglisi E., Cocconcelli P.S. (2015). Understanding the bacterial communities of hard cheese with blowing defect. Food Microbiol..

[122] Agarwal S., Sharma K., Swanson B.G., Yüksel G.U., Clark S. (2006). Nonstarter lactic acid bacteria biofilms and calcium lactate crystals in Cheddar cheese. J. Dairy Sci..

[123] Rosengren A., Fabricius A., Guss B., Sylvén S., Lindqvist R. (2010). Occurrence of foodborne pathogens and characterization of *Staphylococcus aureus* in cheese produced on farm-dairies. Int. J. Food Microbiol..

[124] Linares D.M., Del Río B., Ladero V., Martínez N., Fernández M., Martín M.C., Alvarez M.A. (2012). Factors influencing biogenic amines accumulation in dairy products. Front. Microbiol..

[125] Thommes M., Wang T., Zhao Q., Paschalidis I.C., Segrè D. (2019). Designing metabolic division of labor in microbial communities. mSystems.

[126] Embree M., Liu J.K., Al-Bassam M.M., Zengler K. (2015). Networks of energetic and metabolic interactions define dynamics in microbial communities. Proc. Natl. Acad. Sci. USA.

[127] O’Connor P.M., Kuniyoshi T.M., Oliveira R.P., Hill C., Ross R.P., Cotter P.D. (2020). Antimicrobials for food and feed; a bacteriocin perspective. Curr. Opin. Biotechnol..

[128] Leyva Salas M., Mounier J., Maillard M.B., Valence F., Coton E., Thierry A. (2019). Identification and quantification of natural compounds produced by antifungal bioprotective cultures in dairy products. Food Chem..

[129] Silva C.C.G., Silva S.P.M., Ribeiro S.C. (2018). Application of bacteriocins and protective cultures in dairy food preservation. Front. Microbiol..

[130] Baran R., Brodie E.L., Mayberry-Lewis J., Hummel E., Da Rocha U.N., Chakraborty R., Bowen B.P., Karaoz U., Cadillo-Quiroz H., Garcia-Pichel F. (2015). Exometabolite niche partitioning among sympatric soil bacteria. Nat. Commun..

[131] Koropatkin N.M., Cameron E.A., Martens E.C. (2012). How glycan metabolism shapes the human gut microbiota. Nat. Rev. Microbiol..

[132] Mahony J., McDonnell B., Casey E., van Sinderen D. (2016). Phage-host interactions of cheese-making lactic acid bacteria. Annu. Rev. Food Sci. Technol..

[133] Erkus O., de Jager V.C.L., Spus M., van Alen-Boerritger I.J., van Rijswijck I.M.H., Hazelwood L., Janssen P.W.M., van Hijum S.A.F.T., Kleerebezem M., Smid E.J. (2013). Multifactorial diversity sustains microbial community stability. ISME J..

[134] Rodríguez-Valera F., Martín-Cuadrado A.B., Rodríguez-Brito B., Pasic L., Thingstad T.F., Rohwer F., Mira A. (2009). Explaining microbial population genomics through phage predation. Nat. Rev. Microbiol..

[135] Braga R.M., Dourado M.N., Araújo W.L. (2016). Microbial interactions: Ecology in a molecular perspective. Braz. J. Microbiol..

[136] Pacheco A.R., Segrè D. (2019). A multidimensional perspective on microbial interactions. FEMS Microbiol. Lett..

[137] Smid E.J., Lacroix C. (2013). Microbe-microbe interactions in mixed culture food fermentations. Curr. Opin. Biotechnol..

[138] Irlinger F., Mounier J. (2009). Microbial interactions in cheese: Implications for cheese quality and safety. Curr. Opin. Biotechnol..

[139] Mounier J., Monnet C., Vallaeys T., Arditi R., Sarthou A.S., Hélias A., Irlinger F. (2008). Microbial interactions within a cheese microbial community. Appl. Environ. Microbiol..

[140] Sieuwerts S., de Bok F.A., Hugenholtz J., van Hylckama Vlieg J.E. (2008). Unraveling microbial interactions in food fermentations: From classical to genomics approaches. Appl. Environ. Microbiol..

[141] Iskandar C.F., Cailliez-Grimal C., Borges F., Revol-Junelles A.M. (2019). Review of lactose and galactose metabolism in Lactic Acid Bacteria dedicated to expert genomic annotation. Trends Food Sci. Technol..

[142] Upadhyay V.K., McSweeney P.L.H., Magboul A.A.A., Fox P.F., Fox P.F., McSweeney P.L.H., Cogan T.M., Guinee T.P. (2004). Proteolysis in cheese during ripening. Cheese Chemistry, Physics and Microbiology.

[143] Liu M., Bayjanov J.R., Renckens B., Nauta A., Siezen R.J. (2010). The proteolytic system of lactic acid bacteria revisited: A genomic comparison. BMC Genom..

[144] Noordman W.H., Reissbrodt R., Bongers R.S., Rademaker J.L.W., Bockelmann W., Smit G. (2006). Growth stimulation of *Brevibacterium* sp. by siderophores. J. Appl. Microbiol..

[145] Özcan E., Seven M., Şirin B., Çakır T., Nikerel E., Teusink B., Toksoy Öner E. (2020). Dynamic co-culture metabolic models reveal the fermentation dynamics, metabolic capacities and interplays of cheese starter cultures. Biotechnol. Bioeng..

[146] Monnet C., Back A., Irlinger F. (2012). Growth of aerobic ripening bacteria at the cheese surface is limited by the availability of iron. Appl. Environ. Microbiol..

[147] Schnürer J., Magnusson J. (2005). Antifungal lactic acid bacteria as biopreservatives. Trends Food Sci. Technol..

[148] Arqués J.L., Rodríguez E., Langa S., Landete J.M., Medina M. (2015). Antimicrobial activity of lactic acid bacteria in dairy products and gut: Effect on pathogens. Biomed. Res. Int..

[149] Garnier L., Mounier J., Lê S., Pawtowski A., Pinon N., Camier B., Chatel M., Garric G., Thierry A., Coton E. (2019). Development of antifungal ingredients for dairy products: From *in vitro* screening to pilot scale application. Food Microbiol..

[150] Martinez R.C., Staliano C.D., Vieira A.D., Villarreal M.L., Todorov S.D., Saad S.M., Franco B.D. (2015). Bacteriocin production and inhibition of *Listeria monocytogenes* by *Lactobacillus sakei* subsp. *sakei* 2a in a potentially synbiotic cheese spread. Food Microbiol..

[151] Imran M., Bré J.M., Vernoux J.P., Desmasures N. (2013). Reduced growth of *Listeria monocytogenes* is not associated with individual microbial strains. Food Microbiol..

[152] Loessner M., Guenther S., Steffan S., Scherer S. (2003). A pediocin-producing *Lactobacillus plantarum* strain inhibits *Listeria monocytogenes* in a multispecies cheese surface microbial ripening consortium. Appl. Environ. Microbiol..

[153] McAuliffe O., Hill C., Ross R.P. (1999). Inhibition of *Listeria monocytogenes* in cottage cheese manufactured with a lacticin 3147-producing starter culture. J. Appl. Microbiol..

[154] Ennahar S., Assobhel O., Hasselmann C. (1998). Inhibition of *Listeria monocytogenes* in a smear-surface soft cheese by *Lactobacillus plantarum* WHE 92, a pediocin AcH producer. J. Food Prot..

[155] Núñez M., Rodríguez J.L., García E., Gaya P., Medina M. (1997). Inhibition of *Listeria monocytogenes* by enterocin 4 during the manufacture and ripening of Manchego cheese. J. Appl. Microbiol..

[156] Wan J., Harmark K., Davidson B.E., Hillier A.J., Gordon J.B., Wilcock A., Hickey M.W., Coventry M.J. (1997). Inhibition of *Listeria monocytogenes* by piscicolin 126 in milk and Camembert cheese manufactured with a thermophilic starter. J. Appl. Microbiol..

[157] Delbès-Paus C., Dorchies G., Chaabna Z., Callon C., Montel M.C. (2010). Contribution of hydrogen peroxide to the inhibition of *Staphylococcus aureus* by *Lactococcus garvieae* in interaction with raw milk microbial community. Food Microbiol..

[158] Rilla N., Martínez B., Rodríguez A. (2004). Inhibition of a methicillin-resistant *Staphylococcus aureus* strain in Afuega’l Pitu cheese by the nisin Z-producing strain *Lactococcus lactis* subsp. *lactis* IPLA 729. J. Food Prot..

[159] Ferrari Ida S., de Souza J.V., Ramos C.L., da Costa M.M., Schwan R.F., Dias F.S. (2016). Selection of autochthonous lactic acid bacteria from goat dairies and their addition to evaluate the inhibition of *Salmonella typhi* in artisanal cheese. Food Microbiol..

[160] Garde S., Ávila M., Arias R., Gaya P., Núñez M. (2011). Outgrowth inhibition of *Clostridium beijerinckii* spores by a bacteriocin-producing lactic culture in ovine milk cheese. Int. J. Food Microbiol..

[161] Mathot A.G., Beliard E., Thuault D. (2003). *Streptococcus thermophilus* 580 produces a bacteriocin potentially suitable for inhibition of *Clostridium tyrobutyricum* in hard cheese. J. Dairy Sci..

[162] Rilla N., Martínez B., Delgado T., Rodríguez A. (2003). Inhibition of *Clostridium tyrobutyricum* in Vidiago cheese by *Lactococcus lactis* ssp. *lactis* IPLA 729, a nisin Z producer. Int. J. Food Microbiol..

[163] Bassi D., Gazzola S., Sattin E., Dal Bello F., Simionati B., Cocconcelli P.S. (2020). Lactic acid bacteria adjunct cultures exert a mitigation effect against spoilage microbiota in fresh cheese. Microorganisms.

[164] Murado M.A., Vazquez J.A. (2010). Biphasic toxicodynamic features of some antimicrobial agents on microbial growth: A dynamic mathematical model and its implications on hormesis. BMC Microbiol..

[165] Davies J. (2006). Are antibiotics naturally antibiotics?. J. Ind. Microbiol. Biotechnol..

[166] Herve-Jimenez L., Guillouard I., Guedon E., Boudebbouze S., Hols P., Monnet V., Maguin E., Rul F. (2009). Postgenomic analysis of *Streptococcus thermophilus* cocultivated in milk with *Lactobacillus delbrueckii* subsp. *bulgaricus*: Involvement of nitrogen, purine, and iron metabolism. Appl. Environ. Microbiol..

[167] Callon C., Saubusse M., Didienne R., Buchin S., Montel M.C. (2011). Simplification of a complex microbial antilisterial consortium to evaluate the contribution of its flora in uncooked pressed cheese. Int. J. Food Microbiol..

[168] Bleicher A., Stark T., Hofmann T., Bogovic Matijasić B., Rogelj I., Scherer S., Neuhaus K. (2010). Potent antilisterial cell-free supernatants produced by complex red-smear cheese microbial consortia. J. Dairy Sci..

[169] Imran M., Desmasures N., Vernoux J.P. (2010). From undefined red smear cheese consortia to minimal model communities both exhibiting similar anti-listerial activity on a cheese-like matrix. Food Microbiol..

[170] Monnet C., Bleicher A., Neuhaus K., Sarthou A.S., Leclercq-Perlat M.N., Irlinger F. (2010). Assessment of the anti-listerial activity of microfloras from the surface of smear-ripened cheeses. Food Microbiol..

[171] Maoz A., Mayr R., Scherer S. (2003). Temporal stability and biodiversity of two complex antilisterial cheese-ripening microbial consortia. Appl. Environ. Microbiol..

[172] Liu S.Q., Tsao M. (2009). Inhibition of spoilage yeasts in cheese by killer yeast *Williopsis saturnus* var. *saturnus*. Int. J. Food Microbiol..

[173] Malek R., Bonnarme P., Irlinger F., Frey-Klett P., Onésime D., Aubert J., Loux V., Beckerich J.M. (2018). Transcriptomic response of *Debaryomyces hansenii* during mixed culture in a liquid model cheese medium with *Yarrowia lipolytica*. Int. J. Food Microbiol..

[174] Senaka Ranadheera C., Evans C.A., Adams M.C., Baines (2012). S.K. Probiotic viability and physico-chemical and sensory properties of plain and stirred fruit yogurts made from goat’s milk. Food Chem..

[175] Juillard V., Richard J. (1991). Indirect interaction in milk between proteolytic and isogenic nonproteolytic strains of *Lactococcus lactis*. II. Effect of pre-culturing by a proteolytic strain. Lait.

[176] Baer A., Ryba I. (1995). Influence of casein proteolysis by starter bacteria, rennet and plasmin on the growth of propionibacteria in Swiss-type cheese. Lait.

[177] Sudun W., Arakawa K., Miyamoto M., Miyamoto T. (2013). Interaction between lactic acid bacteria and yeasts in airag, an alcoholic fermented milk. Anim. Sci. J..

[178] Álvarez-Martín P., Flórez A.B., Hernández-Barranco A., Mayo B. (2008). Interaction between dairy yeasts and lactic acid bacteria strains during milk fermentation. Food Control.

[179] Ruiz-Barba J.L., Jiménez-Díaz R. (1995). Availability of essential B-group vitamins to *Lactobacillus plantarum* in green olive fermentation brines. Appl. Environ. Microbiol..

[180] De Freitas I., Pinon N., Maubois J.L., Lortal S., Thierry A. (2009). The addition of a cocktail of yeasts species to Cantalet cheese changes bacterial survival and enhances aroma compound formation. Int. J. Food Microbiol..

[181] Leclercq-Perlat M.N., Corrieu G., Spinnler H.E. (2004). The color of *Brevibacterium linens* depends on the yeast used for cheese deacidification. J. Dairy Sci..

[182] Sieuwerts S., Molenaar D., van Hijum S.A.F.T., Beerthuyzen M., Stevens M.J.A., Janssen P.W.M., Ingham C.J., de Bok F.A.M., de Vos W.M., van Hylckama Vlieg J.E.T. (2010). Mixed-culture transcriptome analysis reveals the molecular basis of mixed-culture growth in *Streptococcus thermophilus* and *Lactobacillus bulgaricus*. Appl. Environ. Microbiol..

[183] Yamauchi R., Maguin E., Horiuchi H., Hosokawa M., Sasaki Y. (2019). The critical role of urease in yogurt fermentation with various combinations of *Streptococcus thermophilus* and *Lactobacillus delbrueckii* ssp. *bulgaricus*. J. Dairy Sci..

[184] Desfossés-Foucault E., LaPointe G., Roy D. (2014). Transcription profiling of interactions between *Lactococcus lactis* subsp. *cremoris* SK11 and *Lactobacillus paracasei* ATCC 334 during Cheddar cheese simulation. Int. J. Food Microbiol..

[185] Viljoen B.C. (2001). The interaction between yeasts and bacteria in dairy environments. Int. J. Food Microbiol..

[186] van den Tempel T., Nielsen M.S. (2000). Effects of atmospheric conditions, NaCl and pH on growth and interactions between moulds and yeasts related to blue cheese production. Int. J. Food Microbiol..

[187] Ponomarova O., Gabrielli N., Sévin D.C., Mülleder M., Zirngibl K., Bulyha K., Andrejev S., Kafkia E., Typas A., Sauer U. (2017). Yeast creates a niche for symbiotic lactic acid bacteria through nitrogen overflow. Cell Syst..

[188] Castellote J., Fraud S., Irlinger F., Swennen D., Fer F., Bonnarme P., Monnet C. (2015). Investigation of *Geotrichum candidum* gene expression during the ripening of Reblochon-type cheese by reverse transcription-quantitative PCR. Int. J. Food Microbiol..

[189] Dugat-Bony E., Straub C., Teissandier A., Onésime D., Loux V., Monnet C., Irlinger F., Landaud S., Leclercq-Perlat M.N., Bento P. (2015). Overview of a surface-ripened cheese community functioning by meta-omics analyses. PLoS ONE.

[190] Pham N.P., Landaud S., Lieben P., Bonnarme P., Monnet C. (2019). Transcription profiling reveals cooperative metabolic interactions in a microbial cheese-ripening community composed of *Debaryomyces hansenii*, *Brevibacterium aurantiacum*, and *Hafnia alvei*. Front. Microbiol..

[191] Mansour S., Bailly J., Landaud S., Monnet C., Sarthou A.S., Cocaign-Bousquet M., Leroy S., Irlinger F., Bonnarme P. (2009). Investigation of associations of *Yarrowia lipolytica*, *Staphylococcus xylosus*, and *Lactococcus lactis* in culture as a first step in microbial interaction analysis. Appl. Environ. Microbiol..

[192] Blaya J., Barzideh Z., LaPointe G. (2018). Symposium review: Interaction of starter cultures and nonstarter lactic acid bacteria in the cheese environment. J. Dairy Sci..

[193] De Pasquale I., Di Cagno R., Buchin S., De Angelis M., Gobbetti M. (2014). Microbial ecology dynamics reveal a succession in the core microbiota involved in the ripening of pasta filata Caciocavallo Pugliese cheese. Appl. Environ. Microbiol..

[194] Settanni L., Moschetti G. (2010). Non-starter lactic acid bacteria used to improve cheese quality and provide health benefits. Food Microbiol..

[195] Rossi F., Marzotto M., Cremonese S., Rizzotti L., Torriani S. (2013). Diversity of *Streptococcus thermophilus* in bacteriocin production; inhibitory spectrum and occurrence of thermophilin genes. Food Microbiol..

[196] Simova E.D., Beshkova D.M., Angelov M.P., Dimitrov Z.P. (2008). Bacteriocin production by strain *Lactobacillus delbrueckii* ssp. *bulgaricus* BB18 during continuous prefermentation of yogurt starter culture and subsequent batch coagulation of milk. J. Ind. Microbiol. Biotechnol..

[197] Medlock G.L., Carey M.A., McDuffie D.G., Mundy M.B., Giallourou N., Swann J.R., Kolling G.L., Papin J.A. (2018). Inferring metabolic mechanisms of interaction within a defined gut microbiota. Cell Syst..

[198] De Filippis F., Genovese A., Ferranti P., Gilbert J.A., Ercolini D. (2016). Metatranscriptomics reveals temperature-driven functional changes in microbiome impacting cheese maturation rate. Sci. Rep..

[199] Fischer C.N., Trautman E.P., Crawford J.M., Stabb E.V., Handelsman J., Broderick N.A. (2017). Metabolite exchange between microbiome members produces compounds that influence *Drosophila* behabiour. eLife.

[200] Acinas S.G., Klepac-Ceraj V., Hunt D.E., Pharino C., Ceraj I., Distel D.L., Polz M.F. (2004). Fine-scale phylogenetic architecture of a complex bacterial community. Nature.

[201] Cosetta C.M., Wolfe B.E. (2019). Causes and consequences of biotic interactions within microbiomes. Curr. Opin. Microbiol..

[202] O’Donnell S.T., Ross R.P., Stanton C. (2020). The progress of multi-omics technologies: Determining function in lactic acid bacteria using a systems level approach. Front. Microbiol..

[203] Castellanos-Rozo J., Pérez Pulido R., Grande M.J., Lucas R., Gálvez A. (2020). Analysis of the bacterial diversity of Paipa cheese (a traditional raw cow’s milk cheese from Colombia) by high-throughput sequencing. Microorganisms.

[204] Gonçalves M.T.P., Benito M.J., Córdoba M.G., Egas C., Merchán A.V., Galván A.I., Ruiz-Moyano S. (2018). Bacterial communities in Serpa cheese by culture dependent techniques, 16S rRNA gene sequencing and high-throughput sequencing analysis. J. Food Sci..

[205] Wolfe B.E., Button J.E., Santarelli M., Dutton R.J. (2014). Cheese rind communities provide tractable systems for in situ and *in vitro* studies of microbial diversity. Cell.

[206] Monnet C., Dugat-Bony E., Swennen D., Beckerich J.M., Irlinger F., Fraud S., Bonnarme P. (2016). Investigation of the activity of the microorganisms in a Reblochon-style cheese by metatranscriptomic analysis. Front. Microbiol..

[207] Valente N.I.P., Rudnitskaya A., Oliveira J.A.B.P., Gaspar E.M.M., Gomes M.T.S.R. (2018). Cheeses made from raw and pasteurized cow’s milk analysed by an electronic nose and an electronic tongue. Sensors.

[208] Zhang Y., Kastman E.K., Guasto J.S., Wolfe B.E. (2018). Fungal networks shape dynamics of bacterial dispersal and community assembly in cheese rind microbiomes. Nat. Commun..

[209] Kastman E.K., Kamelamela N., Norville J.W., Cosetta C.M., Dutton R.J., Wolfe B.E. (2016). Biotic interactions shape the ecological distributions of *Staphylococcus* species. mBio.

[210] Wolfe B.E. (2018). Using cultivated microbial communities to dissect microbiome assembly: Challenges, limitations, and the path ahead. mSystems.

[211] Park W., Yoo J., Oh S., Ham J.S., Jeong S.G., Kim Y. (2019). Microbiological characteristics of Gouda cheese manufactured with pasteurized and raw milk during ripening using Next Generation Sequencing. Food Sci. Anim. Resour..

[212] Wolfe B.E., Dutton R.J. (2013). Towards an ecosystem approach to cheese microbiology. Microbiol. Spectr..

[213] Niccum B.A., Kastman E.K., Kfoury N., Robbat A., Wolfe B.E. (2020). Strain-level diversity impacts cheese rind microbiome assembly and function. mSystems.

[214] Cosetta C.M., Kfoury N., Robbat A., Wolfe B.E. (2020). Fungal volatiles mediate cheese rind microbiome assembly. Environ. Microbiol..

[215] Cosetta C.M., Wolfe B.E. (2020). Deconstructing and reconstructing cheese rind microbiomes for experiments in Microbial Ecology and Evolution. Curr. Protoc. Microbiol..

[216] Tveit A.T., Urich T., Frenzel P., Svenning M.M. (2015). Metabolic and trophic interactions modulate methane production by Arctic peat microbiota in response to warming. Proc. Natl. Acad. Sci. USA.

[217] Tormo J., Barral J. (2004). Accidents de Fromagerie. http://www.accident-fromagerie.fr/spip.php.

[218] Le Bars-Bailly S., Bailly J.D., Brugere H. (1999). Mold-realted failings in cheesemaking. Rev. Med. Vet..

[219] Salazar J.K., Gonsalves L.J., Natarajan V., Shazer A., Reineke K., Mhetras T., Sule C., Carstens C.K., Schill K.M., Tortorello M.L. (2020). Population dynamics of *Listeria monocytogenes*, *Escherichia coli* O157:H7, and native microflora during manufacture and aging of Gouda cheese made with unpasteurized milk. J. Food Prot..

[220] Daly D.F.M., McSweeney P.L.H., Sheehan J.J. (2010). Split defect and secondary fermentation in Swiss-type cheeses—A review. Dairy Sci. Technol..

[221] Sattin E., Andreani N.A., Carraro L., Fasolato L., Balzan S., Novelli E., Squartini A., Telatin A., Simionati B., Cardazzo B. (2016). Microbial dynamics during shelf-life of industrial Ricotta cheese and identification of a *Bacillus* strain as a cause of a pink discolouration. Food Microbiol..

[222] McAuliffe O., Kilcawley K., Stefanovic E. (2019). Symposium review: Genomic investigations of flavor formation by dairy microbiota. J. Dairy Sci..

[223] Alonso R., Picón A., Gaya P., Núñez M. (2013). Proteolysis, lipolysis, volatile compounds and sensory characteristics of Hispanico cheeses made using frozen curd from raw and pasteurized ewe milk. J. Dairy Res..

[224] Topisirovic L., Kojic M., Fira D., Golic N., Strahinic I., Lozo J. (2006). Potential of lactic acid bacteria isolated from specific natural niches in food production and preservation. Int. J. Food Microbiol..

[225] Minty J.J., Singer M.E., Scholz S.A., Bae C.H., Ahn J.H., Foster C.E., Liao J.C., Lin X.N. (2013). Design and characterization of synthetic fungal-bacterial consortia for direct production of isobutanol from cellulosic biomass. Proc. Natl. Acad. Sci. USA.

[226] Ben-Harb S., Saint-Eve A., Panouillé M., Souchon I., Bonnarme P., Dugat-Bony E., Irlinger F. (2019). Design of microbial consortia for the fermentation of pea-protein-enriched emulsions. Int. J. Food Microbiol..

[227] Hu J., Wei Z., Friman V.P., Gu S.H., Wang X.F., Eisenhauer N., Yang T.J., Ma J., Shen Q.R., Xu Y.C. (2016). Probiotic diversity enhances rhizosphere microbiome function and plant disease suppression. mBio.

[228] Vázquez-Castellanos J.F., Biclot A., Vrancken G., Huys G.R., Raes J. (2019). Design of synthetic microbial consortia for gut microbiota modulation. Curr. Opin. Pharmacol..

[229] Carriço J.A., Rossi M., Moran-Gilad J., van Domselaar G., Ramirez M. (2018). A primer on microbial bioinformatics for nonbioinformaticians. Clin. Microbiol. Infect..

[230] Mataragas M., Alessandria V., Ferrocino I., Rantsiou K., Cocolin L. (2018). A bioinformatics pipeline integrating predictive metagenomics profiling for the analysis of 16S rDNA/rRNA sequencing data originated from foods. Food Microbiol..

[231] Parente E., Cocolin L., De Filippis F., Zotta T., Ferrocino I., O’Sullivan O., Neviani E., De Angelis M., Cotter P.D., Ercolini D. (2016). FoodMicrobionet: A database for the visualisation and exploration of food bacterial communities based on network analysis. Int. J. Food Microbiol..

[232] Bockelmann W., Law B.A., Tamime A.Y. (2010). Secondary cheese starter cultures. Technology of Cheesemaking.

[233] Irlinger F., Yung S.A., Sarthou A.S., Delbès-Paus C., Montel M.C., Coton E., Coton M., Helinck S. (2012). Ecological and aromatic impact of two Gram-negative bacteria (*Psychrobacter celer* and *Hafnia alvei*) inoculated as part of the whole microbial community of an experimental smear soft cheese. Int. J. Food Microbiol..

[234] Deetae P., Mounier J., Bonnarme P., Spinnler H.E., Irlinger F., Helinck S. (2009). Effects of *Proteus vulgaris* growth on the establishment of a cheese microbial community and on the production of volatile aroma compounds in a model cheese. J. Appl. Microbiol..

[235] Goerges S., Mounier J., Rea M.C., Gelsomino R., Heise V., Beduhn R., Cogan T.M., Vancanneyt M., Scherer S. (2008). Commercial ripening starter microorganisms inoculated into cheese milk do not successfully establish themselves in the resident microbial ripening consortia of a South German red smear cheese. Appl. Environ. Microbiol..

[236] Feurer C., Vallaeys T., Corrieu G., Irlinger F. (2004). Does smearing inoculum reflect the bacterial composition of the smear at the end of the ripening of a French soft, red-smear cheese?. J. Dairy Sci..

